# Development and preclinical characterization of a novel radiotheranostic EphA2-targeting bicyclic peptide

**DOI:** 10.7150/thno.96641

**Published:** 2024-08-06

**Authors:** Mohamed El Fakiri, Anusha R. Regupathy, Lisa Uhlmann, Nawal Ayada, Nicolas M. Geis, Lisa-Charlotte Domogalla, Johanna Lahdenranta, Ben Blakeman, Francesca Wood, Philipp T. Meyer, Philip Huxley, Matthias Eder, Gemma E. Mudd, Ann-Christin Eder

**Affiliations:** 1 Department of Nuclear Medicine, University Medical Center Freiburg, Faculty of Medicine, University of Freiburg, Hugstetter Str. 55, 79106 Freiburg, Germany.; 2 Division of Radiopharmaceutical Development, German Cancer Consortium (DKTK), partner site Freiburg, Hugstetter Str. 55, 79106 Freiburg, Germany and German Cancer Research Center, Im Neuenheimer Feld 280, 69120 Heidelberg, Germany.; 3 Faculty of Biology, University of Freiburg, 79104 Freiburg, Germany.; 4 BicycleTx Limited, Portway Building, Granta Park, Cambridge CB21 6GS, United Kingdom.; 5 Bicycle Therapeutics, 35 Cambridgepark Drive, Cambridge, MA, 02140, United States.

**Keywords:** theranostics, bicyclic peptide, PET imaging, radionuclide therapy, cancer imaging.

## Abstract

Erythropoietin-producing hepatocellular receptor A2 (EphA2), is a receptor tyrosine kinase involved in cell-cell interactions. It is known to be overexpressed in various tumors and is associated with poor prognosis. EphA2 has been proposed as a target for theranostic applications. Low molecular weight peptide-based scaffolds with low nanomolar affinities have been shown to be ideal in such applications. Bicyclic peptides have emerged as an alternative to traditional peptides for this purpose, offering affinities comparable to antibodies due to their constrained nature, along with high tissue penetration, and improved stability compared to linear counterparts. This study presents the development and comprehensive *in vitro* and *in vivo* preclinical evaluation of BCY18469, a novel EphA2-targeting bicyclic peptide-based radiotheranostic agent.

**Methods:** The EphA2-targeting Bicycle^®^ peptide BCY18469 was identified through phage-display and chemically optimized. BCY18469 was radiolabeled with ^68^Ga, ^177^Lu and ^111^In. The physicochemical properties, binding affinity and internalization as well as specificity of the peptide were evaluated *in vitro*. *In vivo* PET/MR and SPECT/CT imaging studies were performed using [^68^Ga]Ga-BCY18469 and [^111^In]In-BCY18469, respectively, along with biodistribution of [^177^Lu]Lu-BCY18469 up to 24 h post injection in HT1080- and PC-3-tumor bearing BALB/c nu/nu EphA2-overexpressing xenograft mouse models.

**Results:** The EphA2-targeting bicyclic peptide BCY18469 showed high binding affinity toward human and mouse EphA2 (1.9 and 3.8 nM, respectively). BCY18469 specifically bound and internalized into EphA2-expressing HT1080 cells. Imaging studies showed high tumor enrichment at early time-points (SUV of 1.7 g/mL at 1 h p.i. and 1.2 g/mL at 2 h p.i. in PET/MRI, HT1080 xenograft) with tumor contrast as early as 5 min p.i. and kidney-mediated clearance. Biodistribution studies revealed high early tumor uptake (19.5 ± 3.5 %ID/g at 1 h p.i., HT1080 xenograft) with SPECT/CT imaging further confirming these findings (5.7 ± 1.5 %ID/g at 1 h p.i., PC-3 xenograft).

**Conclusion:** BCY18469 demonstrated high affinity, specific targeting of EphA2, a favorable biodistribution profile, and clearance through renal pathways. These findings underscore the potentially important role of bicyclic peptides in advancing radiotheranostic approaches and encourage additional translational research.

## Introduction

Erythropoietin-producing hepatocellular receptor A2 (EphA2) is a transmembrane glycoprotein and part of the receptor tyrosine kinase receptor family 1. As such, it is involved in multiple cellular processes such as cell migration, adhesion, differentiation and death 2. In healthy adult tissues EphA2 expression is generally low 3, but the receptor has been found to be upregulated in numerous solid tumors such as breast, colon, prostate and lung cancer 4-7.

Moreover, expression of EphA2 has been associated with increased carcinogenesis, metastatic disease and poor clinical prognosis 1,8. Therefore, due to its overexpression in cancerous cells, in combination with low expression in healthy tissues, it may represent an ideal target for radiotheranostic applications.

So far, EphA2 has been targeted by non-radioactive-based approaches in both clinical and preclinical settings. Firstly, by inhibition of EphA2 interactions as well as its kinase activity through mainly small molecule drugs 9. Secondly, EphA2 has been used as a target for drug delivery by employing antibody-drug conjugates such as MEDI547, which showed sufficient preclinical efficacy against EphA2-expressing tumors but resulted in non-satisfactory outcomes in a Phase I clinical trial 10,11. Recently, EphA2 has been targeted with bicyclic peptides carrying cytotoxic payloads (Bicycle Toxin Conjugates^®^, BTCs), an approach that showed promising outcomes in preclinical settings and resulted in the identification of BT5528, a BTC^®^ currently being tested in a Phase I/II clinical trial 12-15.

Bicycle peptides have emerged as a promising platform for theranostic approaches 16,17. Bicycle molecules are entropically constrained short peptides, usually around 15 amino acids in length, stabilized in a bicyclic structure using a central chemical scaffold. The constraint imparted by the scaffold allows the achievement of high affinity binding to targets by locking the peptide in its bioactive conformation. Bicycle molecules have a relatively low molecular weight (1.5 - 3 kDa), characterized by rapid tissue penetration and renal excretion, leading to relatively low systemic exposure and favorable accumulation in tissues of interest at early time points 18. Additionally, the bicyclic format can provide enhanced metabolic stability compared to linear peptides, as demonstrated by their resistance to proteolysis 19-21, mainly due to decreased structural flexibility imposed by the rigid rings, as well as through increased intramolecular interactions 16,22. Moreover, the contact surface with protein targets is large compared to the small size of the molecules; leading to increased affinities and excellent target selectivity 23,24. When compared to other molecular entities that have been described previously in the targeting of EphA2 such as antibodies, the pharmacokinetic profile of bicyclic peptides is better suited for systematic application, since the clearance of the antibodies is slow, often requiring days until the optimal accumulation and therefore potentially leading to unwanted increased radiation exposure 25. Small molecule drugs have not been as extensively investigated in targeting the EphA2 receptor. Indeed, only one candidate that also targets EphB4 has been identified, and it did not progress beyond basic initial screenings 26.

Therefore, the properties of bicyclic peptides make them ideal for oncologic radiotheranostic applications aiming at minimized radiation exposure to healthy tissue, accompanied by high and specific tumor uptake. The use of radiolabeled bicyclic peptides (Bicycle Radionuclide Conjugates, BRCs) has already been successfully demonstrated by targeting MT1-MMP (membrane type 1-matrix metalloproteinase) in preclinical imaging settings 17. The BRC [^68^Ga]Ga/[^177^Lu]Lu-BCY-C2 showed high tumor-to-background ratios at early time points combined with fast renal excretion, significantly outperforming a radiolabeled MT1-MMP specific antibody. First-in-human imaging studies using [^68^Ga]Ga-N188, a Bicycle based PET probe which targets nectin-4, highlights the promise of BRCs in clinical applications 27,28.

**Figure 1:**
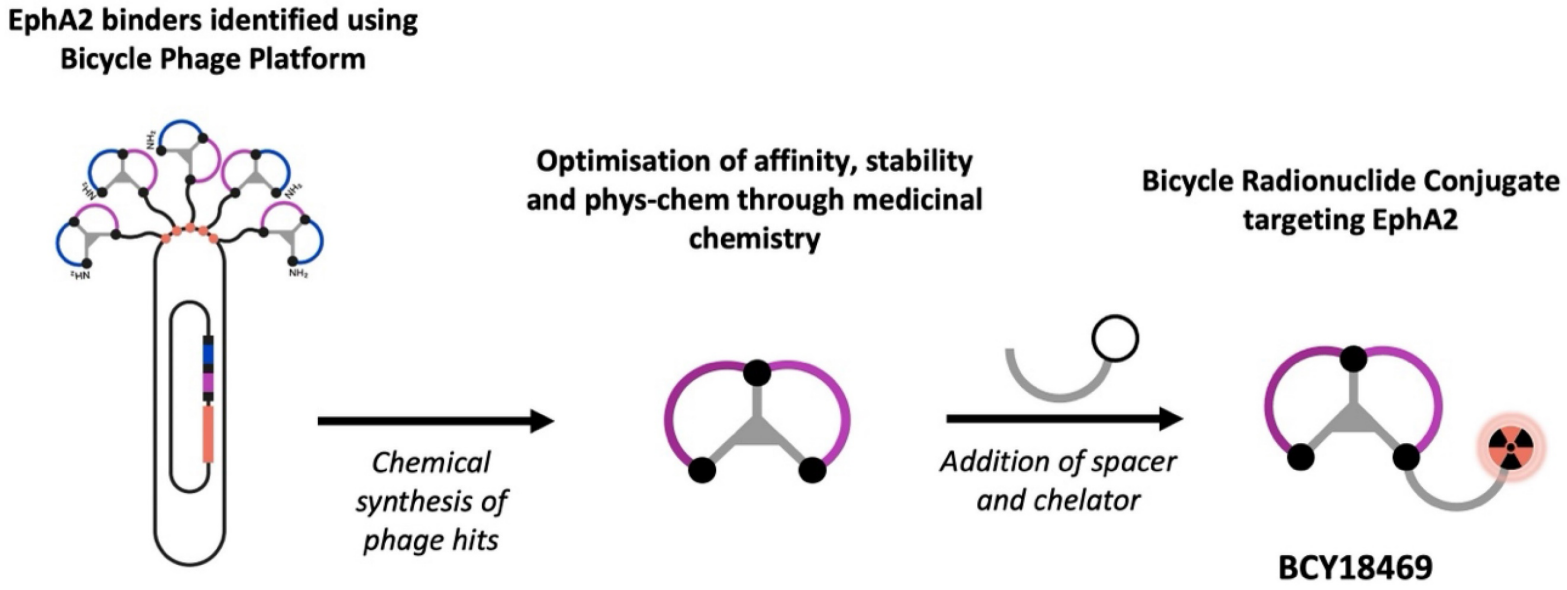
** Schematic representation of the Bicycle Discovery Platform**. Overview of the Bicycle peptide identification platform and its application in the development of novel radiopharmaceuticals.

Following previous work utilizing EphA2-targeting bicyclic peptides for toxin delivery 12, the aim of this study was to assess bicyclic peptides as a tool for EphA2-specific radiotheranostic application. Therefore, previously described bicyclic peptides, identified *via* phage display with high affinity towards EphA2 were employed in this approach. Chemical optimization and introduction of the chelator DOTA resulted in the lead candidate BCY18469, enabling radiolabeling (Figure 1) with the positron-emitting radionuclide gallium-68 (^68^Ga) for PET imaging, the photon-emitting indium-111 (^111^In) for SPECT imaging and the therapeutic β^-^-emitter lutetium-177 (^177^Lu). A full *in vitro* and *in vivo* evaluation of BCY18469 was performed, uncovering the high potential of targeting EphA2 with bicyclic peptides for radiotheranostic applications.

## Materials and Methods

### Chemistry and radiochemistry

Identification and optimization of high affinity EphA2-binding Bicycle peptides was carried out as previously reported 12. BCY18469 and BCY26443 were synthesized using solid phase peptide synthesis and cyclized according to previously described methods 12. For BCY18469, Rink resin (1.85 g, 0.54 mmol/g) was used to generate 1312.4 mg of compound (36.7% yield; MS (ESI): exact mass calcd., 3581.11; found, m/z 1791.5 ([M + 2H]^2+^), 1194.7 ([M + 3H]^3+^); Purity >95% by HPLC). For BCY26443, Rink resin (0.56 g, 0.54 mmol/g) was used to generate 173.0 mg of compound (16.1% yield; MS (ESI): exact mass calcd., 3581.11; found, m/z 1791.3 ([M + 2H]^2+^), 1194.5 ([M + 3H]^3+^). Purity >95% by HPLC) (Supplementary Figures 1, 6 and 7).

^68^Ga was obtained from a commercially available TiO_2_-based ^68^Ge/^68^Ga generator (Eckert & Ziegler Radiopharma GmbH, Berlin, Germany). The generator was eluted with 0.1 M HCl_(aq.)_, the ^68^Ga was then trapped via SCX cartridge and eluted as [^68^Ga]GaCl_3_ for further use in radiolabeling reactions. Lutetium-177 was purchased from Isotope Technologies Munich (ITM, Munich, Germany) as carrier-free [^177^Lu]LuCl_3_ in 0.04 M HCl_(aq.)_. Indium-111 was purchased from Curium (London, UK) as carrier-free [^111^In]InCl_3_ in 0.05 M HCl_(aq.)_.

Radiolabeling with ^68^Ga was performed by mixing equal parts (40 - 150 µL) of 1.0 M HEPES buffer (4-(2-hydroxyethyl)-1-piper-azineethane-sulfonic acid, pH = 4.03) and ^68^Ga-eluate (40 - 150 µL, 30 - 120 MBq), pH of the mixture was adjusted to 4.0 whenever needed by addition of 10 % NaOH_(aq.)_. Thereafter, 2 - 5 nmol (2 - 5 µL of a 1 mM solution) of precursor in DMSO was added. The mixture was heated at 95 °C for 5 minutes, thereafter HPLC and iTLC analyses were carried out (Supplementary Figures 2 and 4). The labeled compound was always used without further purification and only when the amount of free ionic and colloidal ^68^Ga (determined by HPLC and TLC) was < 2%. For *in vivo* applications, the labeling was diluted to the required concentration with 0.9% NaCl and adjusted to pH ∼7 with 30% NaOH_(aq.)_.

Similarly, radiolabeling with ^177^Lu was performed by mixing 50 µL of NH_4_OAc buffer (ammonium acetate, pH = 5.4), 2 - 5 nmol (2 - 5 µL of a 1 mM solution) of precursor in DMSO, and 2 - 15 µL of [^177^Lu]LuCl_3_ (~ 20 - 100 MBq) diluted in 10 - 20 µL of 0.04 M HCl_(aq.)_. The mixture was heated at 95 °C for 15 minutes and analyzed by HPLC and iTLC (Supplementary Figures 3 and 4). Like the ^68^Ga-labeled compound, the ^177^Lu-labeled peptide was used without further purification after dilution to the required concentration with 0.9% NaCl and adjustment of the pH ∼7 with 30% NaOH_(aq.)_.

Indium-111 radiolabeling was performed by adding 65 MBq of [^111^In]InCl_3_ solution into a vial containing 144 µL of 0.1M NH_4_OAc_(aq.)_ buffer (ammonium acetate, pH 5.5) and 1.5 nmol (5 µL of a 0.2 mM solution) of precursor. The reaction was heated at 90 °C with vigorous shaking (500 rpm) for 10 minutes. After HPLC and iTLC analyses (Supplementary Figure 5), the mixture was purified on a preconditioned C18 Sep-Pak cartridge. Preconditioning consisted in flushing with 1 mL EtOH, followed by 5 mL PBS and leaving the cartridge wet. The reaction mixture was diluted with PBS and loaded onto the C18, which was washed with PBS (5 mL). Thereafter, [^111^In]In-BCY18469 was eluted with 200 µL of EtOH/PBS (1:1), followed by 800 µL of PBS for a final volume of 1 mL. Additionally, 5 mg of sodium ascorbate along with 0.05% Tween-20 were added to the final formulation before animal injection.

### Physicochemical properties

For the determination of the lipophilicity (logD_oct/PBS_) of BCY18469, labeling was performed with ^68^Ga (apparent molar activity 1 GBq/µmol). Thereafter an aliquot of 10 µL was added into a 1:1 mixture of *n*-octanol:PBS (500 µL total volume). After shaking and centrifugation at 9,000 rpm for 10 min, a 20-µL fraction of each phase was collected and analyzed in a gamma counter (Wizard2, Perkin Elmer, Germany). Subsequently, the counts of each phase were used to calculate the partition coefficient.

To determine binding to plasma proteins, BCY18469 was labeled with ^68^Ga (apparent molar activity 1 GBq/µmol). Thereafter, 10 µL of the labeling mixture were added to 100 µL of mouse or human plasma (mouse: BioIVT, United Kingdom, human: Sigma Aldrich, Germany). The mixture was left for 15 min at room temperature. Afterwards, the plasma was loaded into an ultrafiltration device (Amicon Ultra 0.5ml - 30 kDa, Merck, Germany) and centrifuged at 14,000 rpm for 30 min at r.t. The filtrate and the membrane were measured in a gamma counter (Wizard2, Perkin Elmer, Germany). The amount of plasma-bound compound was calculated based on the counts in the filtrate relative to the total counts (filtrate + membrane).

Determination of stability in plasma was performed with the ^177^Lu-labeled peptide. 10 µL of the labeled compound were added into human or mouse plasma (mouse: BioIVT, United Kingdom, human: Sigma Aldrich, Germany) and incubated at 37 °C. Aliquots were taken at different time points during incubation (1, 6, 24, 48 and 72 h) to which acetonitrile (30 μL) was added in order to precipitate plasma proteins. After centrifugation at 13,000 rpm for 5 min at r.t. the supernatant was taken and analyzed via HPLC.

### Cell culture

For the *in vitro* evaluation of the novel compound, EphA2-expressing HT1080 (CCL-121, ATCC) and MCF-7 cells (HTB-22, ATCC) were cultured in MEM modified with Earle's Balanced Salt Solution (EMEM, Sigma Aldrich, Germany), non-essential amino acids, 2 mM L-glutamine, 1500 mg/L sodium bicarbonate, 10 % FBS, 1 % sodium pyruvate and 1 % pen/strep. PC-3 cells (CRL-1435, ATCC) were cultured in F-12K medium supplemented with 10 % FBS. Cells were cultured at 37 °C in humidified air supplemented with 5 % CO_2_. When the cells reached a confluence ≥ 80 %, they were harvested using trypsin-EDTA and subsequently processed.

### Binding and internalization properties

#### EphA2 protein production

Human EphA2 (residues 27-529) was cloned into pEXPR-IBA44 between the 5' NheI and 3' BsaI sites to make constructs expressing a BM40 signal sequence and C-terminal 6xHIS tag. For the expression and purification of the protein, HEK293 f/s cells at 1 x 10^6^/mL in freestyle media (Thermo Fisher) were transfected with 1 µg DNA per million cells by PEI either 2:1 or 3:1 PEI:DNA ratio. On day 2 valproic acid was added to a 2.2 mM concentration. The supernatant was harvested 5 days post transfection and loaded on a 1 ml Ni-NTA agarose resin (Thermo Fisher) (wash buffer: 20 mM HEPES pH 7.6, 0.5 M NaCl, 5 mM imidazole, 0.01% Tween-20 buffer; elution buffer: 20 mM HEPES pH 7.6, 0.5 M NaCl, 300 mM imidazole, 0.01% Tween-20). The protein was concentrated to a volume of 2 mL, loaded onto an S-200 Superdex column (GE Healthcare) and eluted with 20 mM HEPES pH 7.6, 150 mM NaCl, 0.01% Tween-20 at a flow rate of 1 mL/min. Fractions were collected and those containing human EphA2 protein were concentrated for further use.

The commercial source details of the recombinant proteins used in SPR were as follows: Human proteins: EphA1 (15789-H02H, Sino Biological), EphA3 (644-A3, R&D Systems), EphA6 (5606-A6, R&D Systems), EphA7 (6756-A7, R&D Systems). Mouse proteins: Mouse EphA2 was purchased from R&D Systems (Product number 639-A2). Mouse EphA2-Fc was resuspended as per manufacturer's recommendations and material was passed through a SuperdexS200 column to remove aggregates.

#### SPR Methodology

SPR experiments were performed on a Biacore 8K+ (Cytiva Life Sciences, USA) instrument, with methods matched for both human and mouse EphA2 protein. Protein was captured on to a Cytiva NTA sensor chip (Cytiva Life Sciences), via the expressed His-tag; this was followed by primary amine coupling chemistry to covalently immobilize the protein to the chip at 25°C. Initially the surface was injected with 0.5 mM NiCl_2_ for 2 minutes, followed by activation of the carboxymethyl dextran surface for 7 minutes using an injection of a 1:1 ratio of 0.4 M 1-ethyl-3-(3-dimethylaminopropyl) carbodiimide hydrochloride (EDC)/0.1 M N-hydroxy succinimide (NHS) at a flow rate of 10 µL/min.

Human EphA2 protein was captured to the Ni-NTA surface to a level of 200-300 RU and Mouse EphA2 to 500-800 RU; the residual activated groups were then blocked with a 7 minute injection of 1 M ethanolamine (pH 8.5):HBS-N (1:1). Finally, the Ni-NTA surface was chelated with a 1-minute injection of 350 mM EDTA.

Peptide was prepared in PBS/0.05% Tween 20 with a final DMSO concentration of 0.5%. The top peptide concentration was 100 or 500 nM and 5 further 3-fold dilutions. The peptide was titrated over the chip using single cycle kinetics at 25°C at a flow rate of 75 µL/min with 60 second association and 4000 second dissociation. All data were double referenced for blank injections and reference surface using standard processing procedures. Data was characterized with a 1:1 kinetic binding model to determine kinetic rate constants ka (M^-1^s^-1^) and kd (s^-1^) and equilibrium dissociation constant KD (nM). All data processing was performed using Cytiva Insight Evaluation (Cytiva Life Sciences, USA).

#### Internalization assay methodology

To assess internalization and specificity, HT1080 cells (10^5^ cells/well) were seeded in a 24-well plate coated with poly-*L*-lysine one day before the experiment. Previous to the experiment, the culture medium was aspirated, the ^68^Ga-labeled compound (apparent molar activity of 2 GBq/µmol) diluted in 250 µL of growth medium + 0.01% Tween 20 (*c* = 30 nM) was added and incubated at 37°C for 45 minutes. To determine specific uptake, 'cold' (non-radiolabeled) BCY18469 was added to half of the wells at an excess concentration of 10 µM/well or, alternatively, the cells were kept at 4 °C during the experiment. Following the incubation, the cells were thoroughly rinsed with cold PBS (3 x 1 mL). Subsequently, surface bound radioactivity was removed by washing with 50 mM glycine (pH 2.8, 2 x 500 µL). Finally, the internalized fraction was determined by lysing the cells with 0.5 M NaOH (500 µL). All fractions were collected and measured using a gamma counter (Perkin Elmer Wizard2, Germany). All counts were standardized to the initial activity, and specific surface binding as well as specific internalization were calculated by subtracting the non-specific binding values obtained from the wells treated with an excess of 'cold' compound. Results were calculated and expressed as %AA/10^5^ cells (% applied activity/10^5^ cells).

### *In vivo* evaluation

#### Pharmacokinetic evaluation

*In vivo* pharmacokinetic studies were conducted in male CD-1 (ICR) mice, 7-9 weeks of age. Three animals were administered 1 mg/kg of BCY18469 in 25 mM Histidine HCl and 10% sucrose (pH 7) by intravenous bolus. Serial venous blood samples were taken at 5 min, 15 min, 30 min, 45 min, 1 h, 2 h and 4 h post injection. Samples were immediately transferred into low-binding microcentrifuge tubes containing 2 μL K2-EDTA (0.5 M) as anti-coagulant and kept on ice until processed by centrifugation at 4°C, 3200 g for 10 min. The resulting plasma was transferred into low-binding tubes and the protein precipitated by addition of methanol containing 0.5% Triton X-100 and internal standards (100 ng/mL each of labetalol, tolbutamide, verapamil, dexamethasone, glyburide and celecoxib). Samples were centrifuged at 4°C, 12000 g for 15 min and the supernatant removed, frozen over dry ice and stored at -70°C until LC-MS/MS analysis (Supplementary Materials - Methods). A calibration curve and quality control samples were prepared in the same matrix. Pharmacokinetic parameters were derived from noncompartmental analysis using the intravenous-noncompartmental model 201 (IV bolus input) in Phoenix WinNonlin version 8.3.5 (Certara, USA).

#### PET/MR imaging of [^68^Ga]Ga-BCY18469

PET/MR imaging was performed in 8-week-old female BALC/c nu/nu mice (Charles River, Germany) which were subcutaneously inoculated on the right upper flank with 10^6^ cells (1:1 in PBS:Matrigel). Tumor growth was monitored, and once size was approx. 300-500 mm^3^, 150 pmol of [^68^Ga]Ga-BCY18469 (4 - 9 MBq) was injected intravenously via tail vein with subsequent imaging performed under isoflurane anesthesia. For the PET imaging experiments with blocking, an excess (15 nmol) of non-radiolabeled BCY18469 was injected 5 mins before the injection of 300 pmol (4 MBq) of the ^68^Ga-labeled compound. PET imaging consisted of a dynamic scan from 0 to 60 min p.i. (30 frames), followed by non-triggered localizer and a 3-step whole body T1-weighed 3D MR-scan (PET/MR 3T, Bruker BioSpin, Germany). Additionally, a 2 h p.i. static image (10 min scan) was recorded, followed by the same MR scan protocol. Data was obtained via ParaVision 3.0 and reconstructed using a Maximum-Likelihood Expectation-Maximization (MLEM) algorithm at 0.5 mm for 18 iterations. Reconstructed data was analyzed with PMOD 3.7.

#### Biodistribution of [^177^Lu]Lu-BCY18469

For the cut and count biodistribution study of [^177^Lu]Lu-BCY18469 the HT1080 xenograft model was used. Once tumors reached a comparable size to the PET imaging study, mice were injected via the tail vein with 150 pmol of the ^177^Lu-labeled bicyclic peptide and sacrificed at 1, 2, 6 and 24 h p.i. (n = 3 mice per time point). Thereafter, mice were dissected, and organs of interest harvested, which included: blood, heart, lung, spleen, liver, kidneys, muscle (quadriceps), intestine, brain, tail, bone (femur) and grafted tumor.

#### SPECT/CT imaging of [^111^In]In-BCY18469

SPECT/CT imaging was performed in three 5- to 8-week-old male athymic nude mice (Janvier Labs, UK) which were subcutaneously inoculated on the right upper flank with 5 x 10^6^ PC-3 cells (1:1 in PBS:Matrigel). Tumor growth was monitored, and once size was approx. 200 - 500 mm^3^, mice were injected intravenously via tail vein with 230 pmol of [^111^In]In-BCY18469 (5.3 ± 0.2 MBq). Mice were imaged under isoflurane anesthesia using whole-body static SPECT followed by a CT at each timepoint (30 min SPECT scan time starting at 1h and 24h p.i.). Reconstructed images were generated in units of activity. Namely, the values assigned to the voxels (volume elements) comprising the 3D reconstructed SPECT images were in units of MBq or equivalent. Reconstructed images were processed with VivoQuant (Invicro). Whole body maximum intensity projection (MIP) images were generated using VivoQuant.

Animal experiments were performed according to the directive 2010/63/EU of the European Parliament and the European Council on the protection of animals used for scientific purposes. Experiments were approved by the Federal Republic of Germany, Regional Council Freiburg (G-17/142 and G-23/077) or according to United Kingdom's Guidance on the Operation of Animals (Scientific Procedures) Act of 1986, under Home Office Project License number PP6127261.

### Statistics

All experiments were performed at least in triplicate, with exception of the PET/MR imaging (*n* = 1) and SPR experiments (*n* = 2). All multiple measurement results are expressed as value ± SD. Data was analyzed and plotted using Prism (version 8.0.1, GraphPad Software), when applicable *P* values were determined by Student's *t*-test and were considered significant when smaller than 0.05.

## Results

### Chemistry and Radiochemistry

*De novo* identification of a novel EphA2 binding Bicycle series was performed as previously described 12. Nine phage libraries with random residues between the three bicyclic peptide-linking cysteine residues were panned against EphA2 using a hydrophilic TATA scaffold. From this panning, six distinct chemical families were identified. The abundantly occurring sequences were submitted to affinity maturation, giving a lead series with high affinity for EphA2 (K_i_ < 10 nM). Chemical modifications to increase the affinity, hydrophilic character and stability of the Bicycle series were performed, followed by incorporation of a hydrophilic Sarcosine10 spacer and DOTA chelator. This gave BRC BCY18469, which showed high affinity binding (K_D_ = 1.93 nM) to EphA2 and was progressed to further *in vitro* and *in vivo* profiling.

In regards to radiochemistry, labeling of BCY18469 with ^68^Ga, ^111^In and ^177^Lu resulted in radiochemical conversions (RCCs) ≥ 98 % as shown by HPLC and TLC (Supplementary Figures 2 to 5). RCC was determined following consensus guidelines 29.

### *In vitro* evaluation

Determination of physicochemical properties of BCY18469 confirmed its hydrophilic nature with a remarkably low log*D*_pH 7.4_ of -3.57 in *n-*octanol/PBS (Table 1). Furthermore, [^68^Ga]Ga-BCY18469 showed moderate binding to human and mouse plasma proteins as well as high serum stability, with 94 % of peptide remaining intact after 72 h in human plasma and 40 % in mouse (Supplementary Figure 11).

BCY18469 binds with high affinity to recombinant human EphA2, as determined by surface plasmon resonance (SPR) (K_D_ 1.93 ± 0.35 nM). Importantly, cross reactivity with recombinant mouse EphA2 protein was also demonstrated (K_D_ 3.82 ± 0.56 nM), allowing for the detection of any EphA2 mediated uptake in non-tumor tissue (Table 1, Supplementary Figure 8 and Supplementary Table 1). BCY18469 shows a high degree of selectivity, with no binding observed to a panel of closely related homologues of EphA2 when assessed by SPR. A non-binding control BRC BCY26443 showed no binding to human EphA2 by SPR (Supplementary Figure 8, Supplementary Table 1).

**Figure 2:**
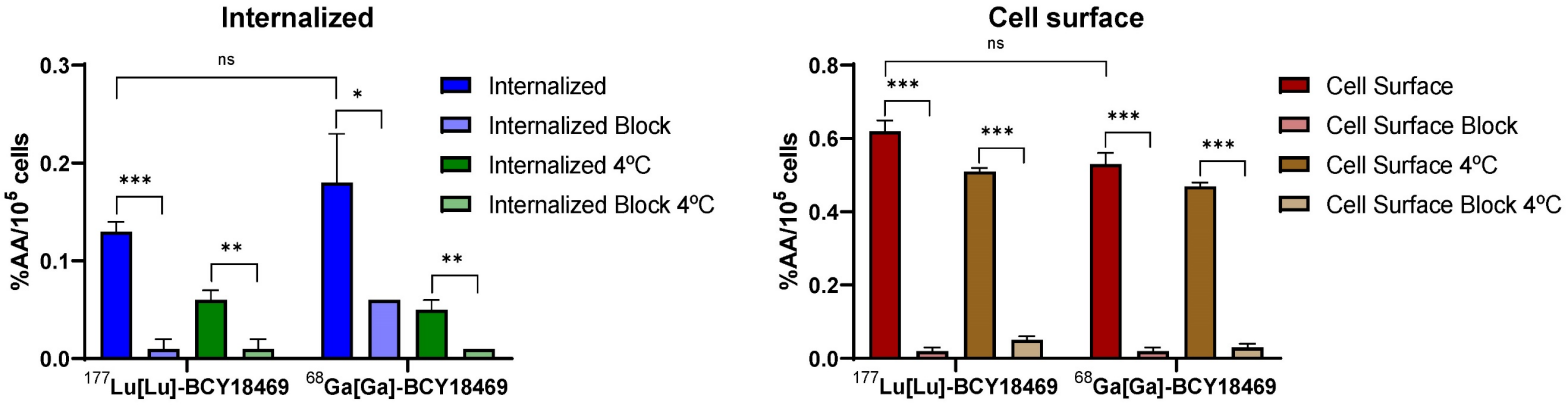
** Cell surface binding and internalization of ^68^Ga/^177^Lu-labeled BCY18469.** Specificity of internalization was determined by blocking with an excess of non-labeled compound (10 µM) and by incubation at 4 °C. Data expressed as % applied activity/10^5^ cells (n = 3). ns = non-significant, * = p < 0.05; ** = p < 0.01; *** = p < 0.001.

Internalization experiments of [^68^Ga]Ga-BCY18469 revealed specific binding towards HT1080 cells 45 min post incubation (Figure 2). The uptake was proven to be specific by blocking with an excess of non-labeled peptide, the difference between blocked and non-blocked values was found to be statistically significant (*p* < 0.05). Additionally, internalization at 4 °C, a temperature in which endocytosis processes are hampered, was decreased and similar to blocked uptakes at 37 °C, confirming the specificity of the binding. Further experiments with the non-binding bicycle [^68^Ga]Ga-BCY26443 also showed no cell uptake (Supplementary Figure 12). Additionally, there was no significant difference between the cell uptake values of the ^177^Lu-labeled and ^68^Ga-labeled BCY18469 (*p* > 0.05).

### *In vivo* evaluation

PK parameters of BCY18469 were assessed following intravenous bolus administration to mice at 1 mg/kg. Relatively high plasma clearance (24 ml/min/kg, greater than glomerular filtration) and low volume of distribution at steady state (0.39 L/kg) were observed, resulting in a short terminal half-life (0.24 h). All parameters can be found in the Supplementary Table 7.

**Figure 3:**
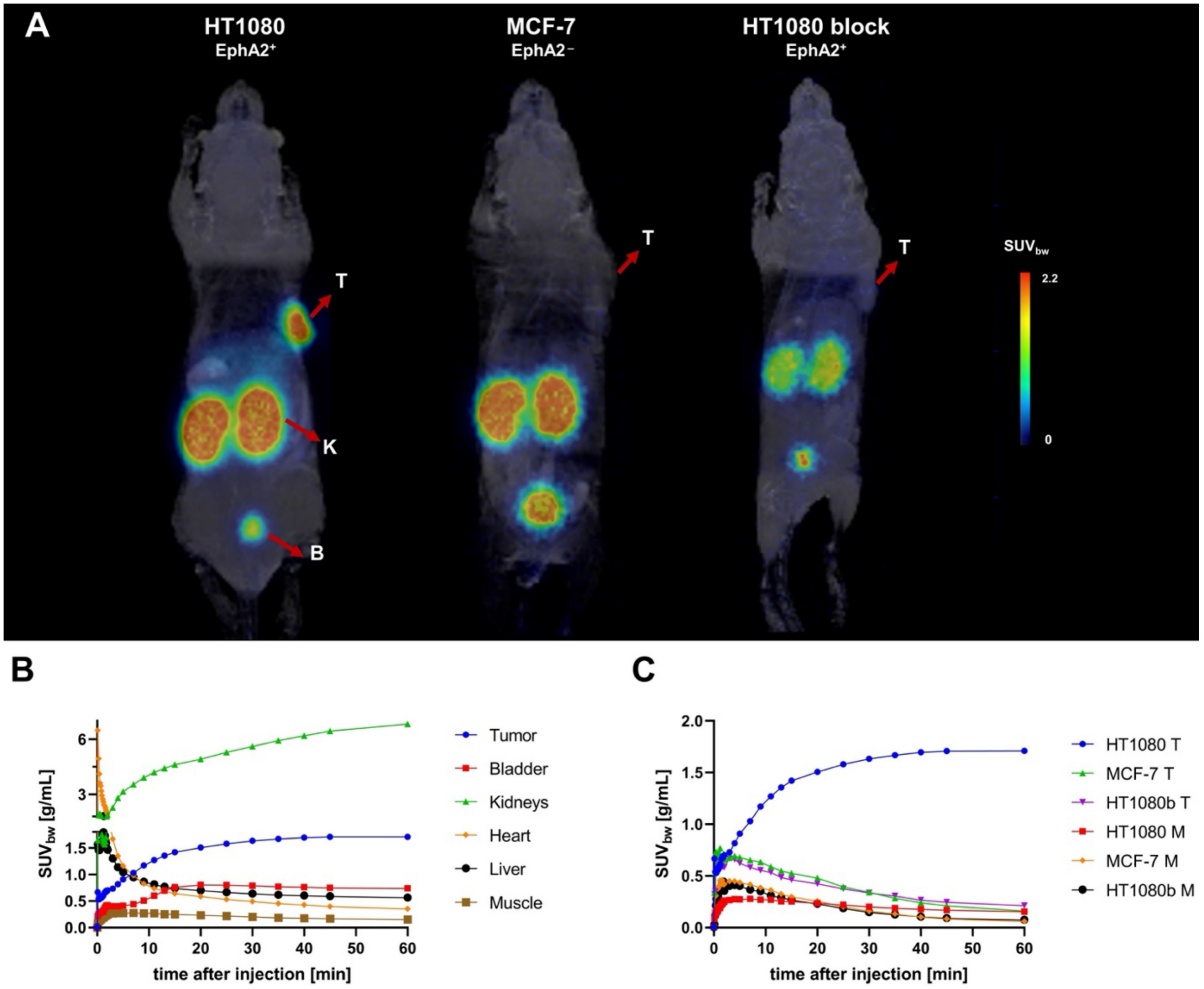
** PET/MRI imaging of [^68^Ga]Ga-BCY18469 in tumor xenograft mouse models**.** A)** PET/MR Maximum Intensity Projection (MIP) 2 h p.i. of 150 pmol of [^68^Ga]Ga-BCY18469 in an HT1080 (left) and MCF-7 xenograft (middle), and 2 h p.i. of 300 pmol of [^68^Ga]Ga-BCY18469 after 15 nmol of BCY18469 in an HT1080 xenograft (right). T: tumor, K: kidney, B: bladder; **B)** Time-activity-curve (TAC) for organs of interest from 0 to 60 min p.i. of 150 pmol of [^68^Ga]Ga-BCY18469 in the HT1080 xenograft; **C)** Detailed TACs of tumor (T) and muscle (M, background).

**Figure 4:**
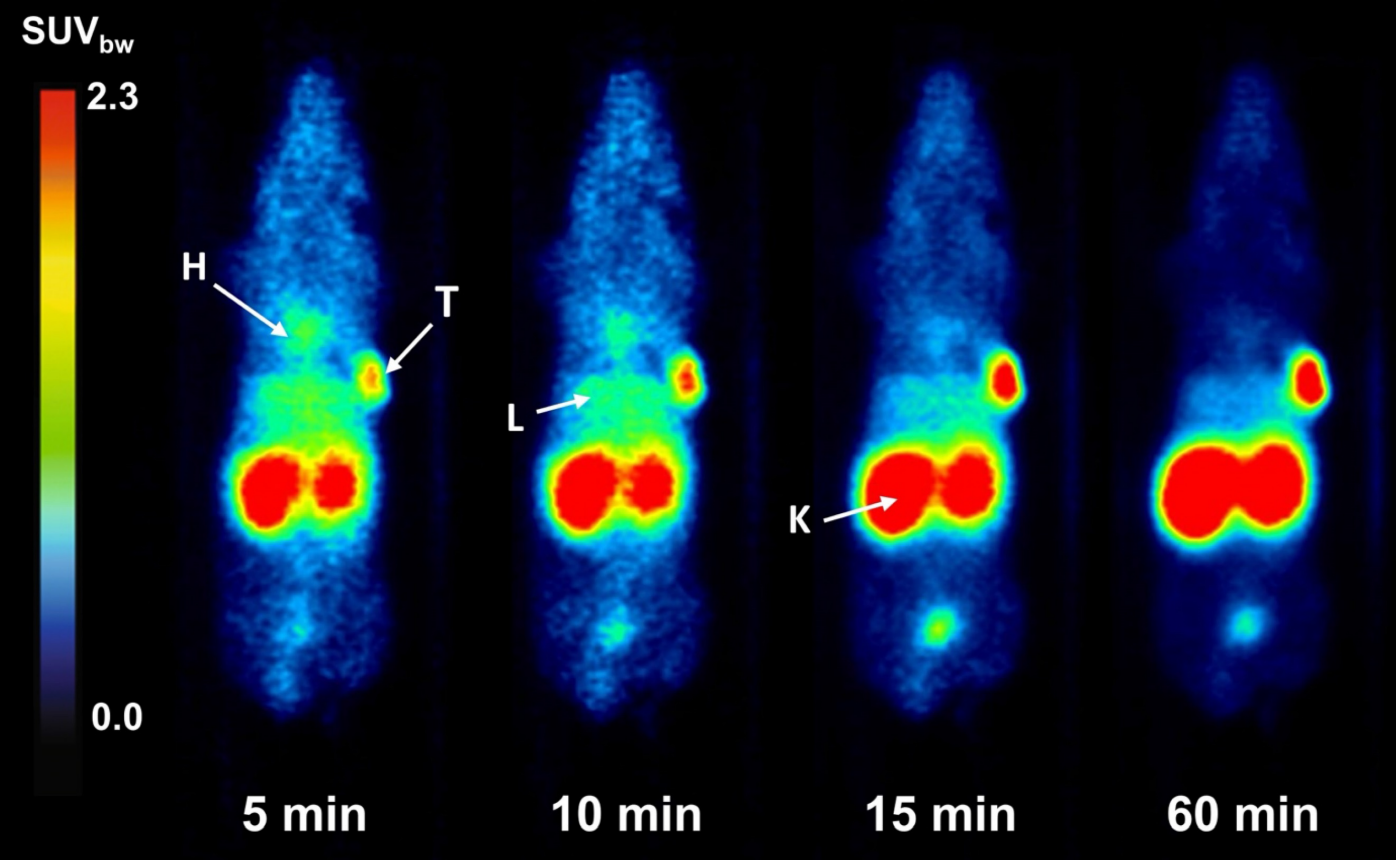
**Coronal slices of dynamic PET imaging of [^68^Ga]Ga-BCY18469 in a HT1080 tumor xenograft mouse model.** Imaging at various time points after injection of 150 pmol (9 MBq) [^68^Ga]Ga-BCY18469. **H**: heart; **T**: tumor; **L**: liver; **K**: kidney.

PET/MR imaging of [^68^Ga]Ga-BCY18469 in HT1080 mouse xenograft showed high tumor accumulation (SUV_bw_ of 1.7 g/mL at 1 h p.i. and 1.2 g/mL at 2 h p.i. ) along with rapid clearance of non-tumor bound peptide through renal pathways as shown by the time-activity-curve (Figure 3). Tumor-to-muscle ratio at 1 h p.i. and 2 h p.i. proved very high imaging contrast, which is further demonstrated by the maximum intensity projection images and coronal slices (Figures 3 and 4, Supplementary Tables 2 and 3). Imaging of the EphA2-negative MCF-7 xenograft showed no uptake of ^68^Ga-labeled BCY18469 in the tumor proving the compounds' specificity. This finding was confirmed by PET imaging in the HT1080 xenograft when blocking with an excess of non-labeled compound. Additionally, imaging with the non-binding [^68^Ga]Ga-BCY26443 further proved specificity (Supplementary Figure 14).

**Figure 5:**
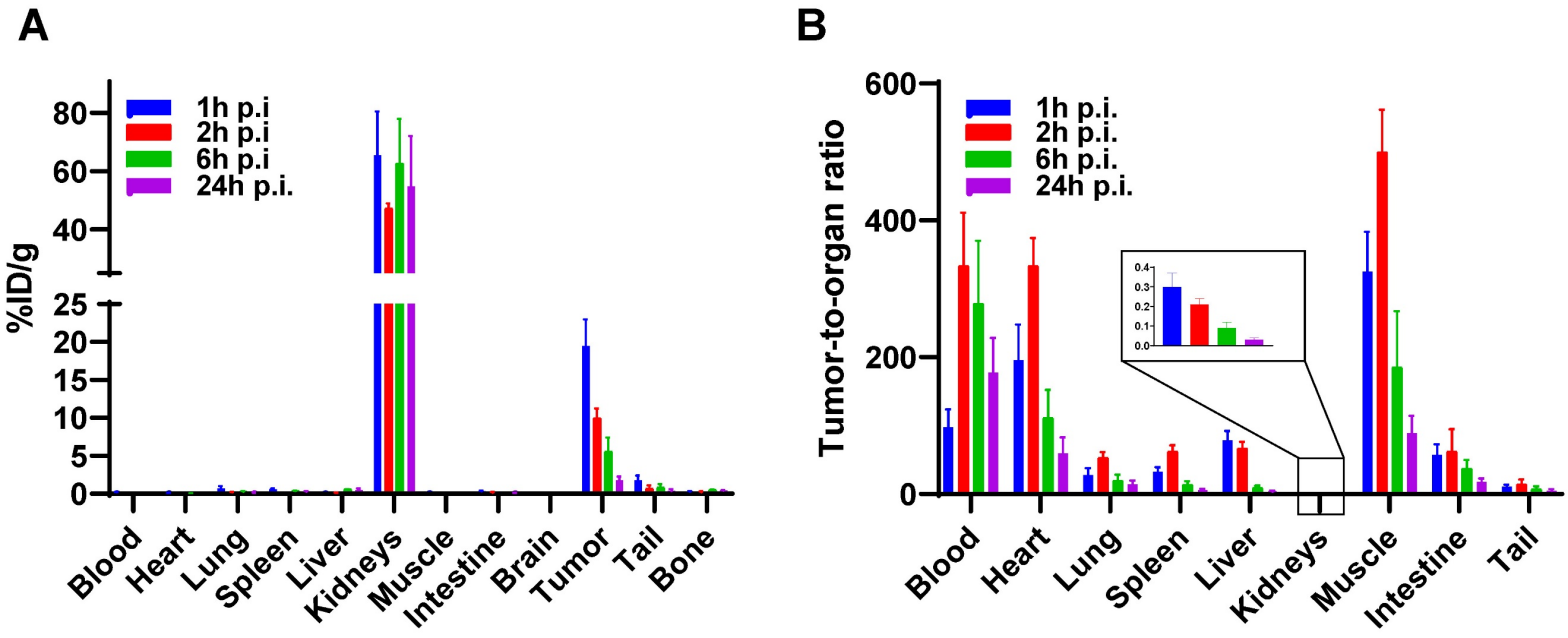
** Biodistribution of [^177^Lu]Lu-BCY18469 in HT1080 tumor-bearing nude mice. A)** After injection of 150 pmol of peptide at 1, 2, 6 and 24 h p.i. **B)** Tumor-to-organ ratios at 1, 2, 6 and 24 h p.i. Data expressed as mean %injected dose(ID)/g tissue ± SD (n = 3)

A biodistribution study of [^177^Lu]Lu-BCY18469 confirmed the PET/MRI findings. Tumor enrichment within 1 h p.i. was high with 19.50 ± 3.50 %ID/g (Figure 5, Supplementary Tables 4 and 5). The tumor uptake decreased over time with 5.56 ± 1.24 %ID/g at 6 h p.i. and 1.77 ± 0.51 %ID/g at 24 h p.i. Non-target organs showed negligible uptake (≤ 1 %ID/g) throughout all time points. Due to the renal excretion pathway [^177^Lu]Lu-BCY18469 uptake in kidneys persisted high with 54.86 ± 17.12 %ID/g at 24 h p.i.

**Figure 6:**
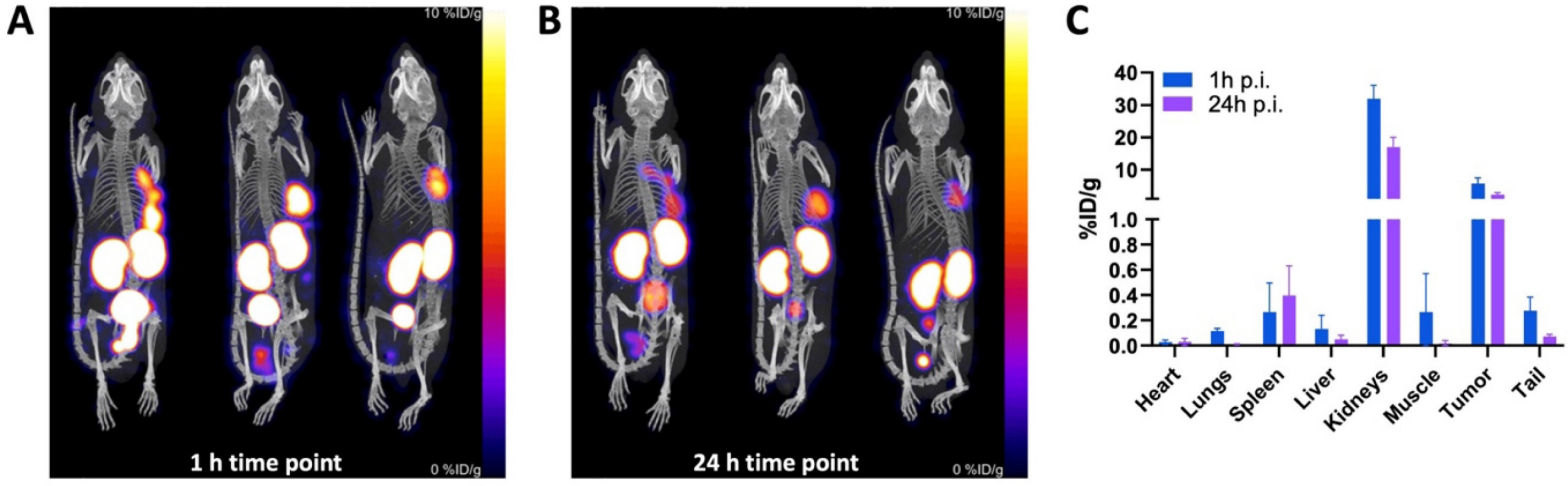
** SPECT/CT imaging of [^111^In]In-BCY18469 in a PC-3 xenograft model. A)** Maximum intensity projection at 1 h and **B)** 24 h p.i. of 230 pmol (5.3 ± 0.2 MBq) of [^111^In]In-BCY18469. **C)** Quantified data (%ID/g) of [^111^In]In-BCY18469 SPECT imaging.

SPECT/CT imaging with [^111^In]In-BCY18469 in PC-3 tumor bearing mice was in line with the previous findings by PET and biodistribution experiments in HT1080 tumor bearing mice (Figure 6). SPECT imaging at 1 h p.i. further confirmed the good tumor uptake at early time-points with 5.72 ± 1.47 %ID/g. At 24 h p.i., it showed sufficient contrast and an uptake of 2.32 ± 0.50 %ID/g. Furthermore, non-target organs had negligible uptakes (≤ 1 %ID/g) and the excretion through renal pathways was comparable to the PET findings at 1 h p.i. (31.9 ± 3.4 %ID/g in kidney). Detailed values can be found in the Supplementary Table 6.

## Discussion

The development of novel tumor-targeting radiopharmaceuticals both for oncological molecular imaging and for therapeutic applications is of high clinical relevance 30,31. Additionally, new receptors that can be targeted across a spectrum of cancerous diseases are of utmost importance to diversify treatment options and improve personalized care. This is exemplified by e.g. FAP-targeting radiopharmaceuticals, which are some of the most recent 'pan-cancer' targeting vectors with potential to expand the spectrum of oncologic radiotheranostics 32. The expression of EphA2 in various solid tumors has made it attractive for targeting in molecular imaging approaches. EphA2 has been targeted by various approaches through radiolabeled antibody and antibody-like biomolecules 33,34. Most interestingly, Burvenich *et al.* reported successful EphA2 imaging in a xenograft model through a zirconium-89 labeled anti-EphA2 antibody 35. The same ^89^Zr-labeled antibody was tested in a Phase I study, concluding that its tumor penetration was poor and not sufficient for clinical applications 36. Moreover, efforts have been made with a ^99m^Tc-labeled phage display-derived SWL peptide, resulting in relatively low tumor uptakes 37. More recently, bicyclic peptides have been exploited, which offer advantages over mAb's and linear peptides, while maintaining high selectivity and affinity towards the target. In this regard, the bicyclic peptide [^68^Ga]Ga-BCY6164 was the first reported proof-of-concept BRC to image EphA2 in an HT1080-bearing mouse xenograft 12. Moreover, inspired by previous work, Gan *et al.* recently reported imaging on the EphA2-expressing PC3 cell line in a murine model, with an [^18^F]AlF-NOTA-labeled version of BCY6164 38.

Considering the promising performance of bicyclic peptides in radiotheranostic approaches, a novel EphA2-targeting bicyclic peptide was developed through phage display aiming at an optimization for future clinical translation as a radiotheranostic agent. The best candidate from successive rounds of panning was affinity matured and modified chemically to further increase its affinity and proteolytic stability. After introduction of the metal-chelating moiety DOTA, BCY18469 was identified as the lead candidate with nanomolar affinity towards human EphA2 (K_D_ = 1.93 ± 0.35 nM) and good cross reactivity with mouse EphA2 (K_D_ = 3.82 ± 0.56 nM).

BCY18469 was successfully radiolabeled with three clinically relevant radionuclides, ^68^Ga, for PET, ^177^Lu for therapy and ^111^In for SPECT. The radiolabeling under the described conditions did not result in any degradation; demonstrating the chemical robustness of this bicyclic peptide. Additionally, [^177^Lu]Lu-BCY18469 showed minimal degradation in human plasma over 72 hours, following chemical optimization aiming to reduce metabolic susceptibility. Although [^177^Lu]Lu-BCY18469 was degraded to a greater degree in mouse plasma over 72 hours, with 40% of parental compound remaining, it was deemed to be acceptable given the typically short circulating half-life of bicyclic peptides, as exemplified by the PK studies of BCY18469 (t_½_ = 0.24 h). Furthermore, [^68^Ga]Ga-BCY18469 showed modest plasma protein binding of around 50%, which is consistent with the rapid clearance observed in the *in vivo* biodistribution and PK studies.

Further *in vitro* testing of BCY18469 ensured high affinity and specificity towards EphA2 in both isolated protein and cell-bound receptor. These results were comparable to recently reported ^18^F-labeled bicyclic peptides (~ 0.2 % AA/10^5^ cells) although work was performed in PC-3 cells, which expresses EphA2 in a higher degree compared to HT1080 6,39. The expression of EphA2 in various cell lines has been extensively evaluated by FACS in previous work 13.

The promising *in vitro* performance of both the ^68^Ga and ^177^Lu-labeled versions of BCY18469 allowed for its progression into preclinical *in vivo* testing. [^68^Ga]Ga-BCY18469 was used in PET-imaging studies in an HT1080 mouse xenograft. PET/MRI revealed fast renal elimination, resulting in sufficient imaging contrast as early as 5 minutes p.i. At 2 h p.i. a static scan confirmed the findings from the dynamic imaging, proving high uptake of [^68^Ga]Ga-BCY18469 in the tumor at early time points, as well as high tumor-to-background contrast. The *in vivo* specificity of BCY18469 towards EphA2 was proven by both PET imaging in an EphA2-negative cell line (MCF-7) and blocking with an excess on non-radiolabeled BCY18469, with both experiments showing no significant uptake and no tumor-to-background contrast (Figure 3). Imaging with the non-binder [^68^Ga]Ga-BCY26443 as an additional negative control further proved the specificity of BCY18469.

To further confirm the data from the PET/MR imaging, biodistribution studies with [^177^Lu]Lu-BCY18469 were performed in the same mouse xenograft model. A high tumor uptake at 1- and 2-hour p.i. was observed, while after 24 hours a significant decrease in the retained activity was detected. This decrease in tumor retained activity at later time points correlates with the relatively low internalization ratios determined in the *in vitro* assays. Low internalization leads to the majority of the peptide being surface-bound and not able to enter the tumor cell, allowing faster washout of the activity. Further modifications of BCY18469 to increase even more its affinity towards EphA2 or its contact surface area with the receptor, could potentially improve the tumor retention over time. Notably, the majority of non-tumor bound radioactivity was cleared rapidly, with no activity detected in any other organ than kidney by 1 h p.i. Tumor-to-background (muscle) ratios were high through all time points with an impressively high ratio of 325 at 1 h p.i. and ending with a ratio of 88 at 24 h p.i.

SPECT imaging with [^111^In]In-BCY18469 was also in line with the findings in the PET and biodistribution studies, and further confirmed the high accumulation of BCY18469 in EphA2-overexpressing tumor tissue as well as the rapid tumor uptake and clearance. Notably, the SPECT/CT imaging was carried out employing the PC-3 cell line as an alternative to the HT1080. This allowed for the validation of BCY18469 in an additional cell line, with satisfactory and comparable results, confirming the positive performance of the bicyclic peptide across cell lines.

Nevertheless, through all the *in vivo* experiments, a notable fraction of activity was retained in the kidneys, a phenomenon that is known for peptide-based radiopharmaceuticals which are renally cleared. However, an additional reason for the persistence of BCY18469 in the kidneys could be the peptide binding non-specifically to amino acid transporters and/or ion transporters present in the kidney and its proximal tubes, which might be addressed by e.g. administration of gelofusine 40-42. Furthermore, the obtained results are also in line with previous findings on tumor-targeting bicyclic peptides 12,17,38.

The promising performance of BCY18469 in detecting EphA2-positive tumors in preclinical settings postulates it as an encouraging candidate for clinical translation in imaging applications with short-lived radionuclides, especially in cancer entities where specific biomarkers for molecular imaging have yet not been discovered e.g. pancreatic, gastric/ esophageal, non-small cell lung and head and neck cancers. In addition, the implication of having sufficient imaging contrast, in a mouse xenograft, as early as 5 minutes p.i. could significantly increase outputs and reduce given doses in clinical imaging practices. Moreover, various approaches could be explored to optimize the biodistribution profile of this bicyclic peptide series 42.

## Conclusion

In this study, BCY18469 was identified via phage display and was chemically optimized to give a high affinity, biologically stable bicyclic peptide targeting EphA2. The bicyclic peptide was radiolabeled with clinically relevant radionuclides and evaluated both *in vitro* and *in vivo*. BCY18469 showed high affinities, EphA2 specificity, and a favorable biodistribution profile with excellent tumor targeting properties at early timepoints and renal-mediated clearance. These results highlight the potential of bicyclic peptides in radiotheranostic approaches and grant further translational research.

## Supplementary Material

Supplementary methods, figures and tables.

## Figures and Tables

**Table 1:** Physicochemical and *in vitro* characterization data of BCY18469. **^a^** Human plasma_;_ **^b^** Mouse plasma, ^c^Human EphA2, ^d^Mouse EphA2

Parameter	BCY18469	
Mass (MALDI-TOF) (m/z [M+H]^+^)	3582.22 (calc. 3281.1)	
Lipophilicity (logD_oct/PBS_)	- 3.57 ± 0.17	
Plasma protein binding (% bound peptide)	43.3 ± 4.0 % **^a^**	51.1 ± 1.4 % **^b^**
Affinity (K_D_ - SPR)	1.93 ± 0.35 nM ^c^	3.82 ± 0.56 nM ^d^
