# Supplementary material for: Development and preclinical characterization of a novel radiotheranostic EphA2-targeting bicyclic peptide
Source: Theranostics. 2024 Aug 6;14(12):4701–12. doi: 10.7150/thno.96641 (PMC11373624; doi:10.7150/thno.96641)
Supplement: Supplementary file 1 — Supplementary methods, figures and tables. [file thnov14p4701s1.pdf]

Supplementary Material

Development and Preclinical Characterization of a Novel Radiotheranostic EphA2-targeting Bicyclic Peptide

Mohamed El Fakiri[1,2,3], Anusha R. Regupathy[4], Lisa Uhlmann[1,2], Nawal Ayada[1,2], Nicolas M. Geis[1,2,3], Lisa-Charlotte Domogalla[1,2], Johanna Lahdenranta[5], Ben Blakeman[4], Francesca Wood[4], Philipp T. Meyer[1], Philip Huxley[4], Matthias Eder[1,2], Gemma E. Mudd[4], Ann-Christin Eder[1,2]

1. Department of Nuclear Medicine, University Medical Center Freiburg, Faculty of Medicine, University of Freiburg, Hugstetter Str. 55, 79106 Freiburg, Germany.
2. German Cancer Consortium, Partner Site Freiburg, Hugstetter Str. 55, 79106 Freiburg, Germany, and German Cancer Research Center, Im Neuenheimer Feld 280, 69120 Heidelberg, Germany
3. Faculty of Biology, University of Freiburg, 79104 Freiburg, Germany
4. BicycleTx Limited, Portway Building, Granta Park, Cambridge CB21 6GS, United Kingdom
5. Bicycle Therapeutics, 35 Cambridgepark Drive, Cambridge, MA, 02140, United States

Table of Contents

S1. Supplementary Methods 2

S2. Supplementary Figures 3

Supplementary Figure 1. HPLC analysis of BCY18469 and BCY26443 3
Supplementary Figure 2. HPLC UV radio HPLC of [^{68}Ga]Ga-BCY18469 4
Supplementary Figure 3. Radio HPLC of [^{177}Lu]Lu-BCY18469 4
Supplementary Figure 4. Radio iTLC analysis of [^{68}Ga]Ga-BCY18469 and [^{177}Lu]Lu-BCY18469 5
Supplementary Figure 5. Phosphor image of radio iTLC and HPLC of [^{111}In]In-BCY18469 ... 5
Supplementary Figure 6. MALDI-TOF spectrum of BCY18469 5
Supplementary Figure 7. ESI-MS spectrum of BCY18469 and BCY26443 6
Supplementary Figure 8. Exemplary SPR sensograms of BCY18469 and binding results for the EphA2 non-binder BCY26443. 7
Supplementary Figure 9. Plasma concentration–time profile of BCY18469 8
Supplementary Figure 10. Chemical structure of BCY18469 and BCY26443. 9
Supplementary Figure 11. In vitro serum stability of [^{177}Lu]Lu-BCY18469 10
Supplementary Figure 12. Cell surface binding and internalization of [^{68}Ga]Ga-BCY26443 10
Supplementary Figure 13. PET/MR Maximum Intensity Projection 1 h p.i. of [^{68}Ga]Ga-BCY18469 in an HT1080, MCF-7 xenograft, and in an HT1080 xenograft with block 11
Supplementary Figure 14. PET/MR Maximum Intensity Projection 1 h and 2 h p.i. of non-EphA2 binding [^{68}Ga]Ga-BCY26443 in an HT1080 mouse xenograft. 11

S3. Supplementary Tables 12

Supplementary Table 1. Affinity values of BCY18469 obtained from SPR 12
Supplementary Table 2. Detailed internalization values for ^{68}Ga and ^{177}Lu-labeled BCY18469 12
Supplementary Table 3. Uptake values of [^{68}Ga]Ga-BCY18469 over 60 min p.i. (in SUV_{bw}) in PET/MRI after injection of 150 pmol in an HT1080 tumor xenograft. 12
Supplementary Table 4. Uptake values of [^{68}Ga]Ga-BCY18469 over 60 min p.i. (in %ID/g) in PET/MRI after injection of 150 pmol in an HT1080 tumor xenograft. 13
Supplementary Table 5. Uptake values of [^{68}Ga]Ga-BCY18469 over 60 min p.i. (in SUV_{bw}) in PET/MRI after injection of 150 pmol in a MCF-7 tumor xenograft 14
Supplementary Table 6. Uptake values of [^{68}Ga]Ga-BCY18469 over 60 min p.i. (in %ID/g) in PET/MRI after injection of 150 pmol in a MCF-7 tumor xenograft 15
Supplementary Table 7. Uptake values of [^{68}Ga]Ga-BCY18469 over 60 min p.i. (in SUV_{bw}) in PET/MRI after injection of 300 pmol after 15 nmol of non-labeled compound in an HT1080 tumor xenograft. 16
Supplementary Table 8. Uptake values of [^{68}Ga]Ga-BCY18469 over 60 min p.i. (in %ID/g) in PET/MRI after injection of 300 pmol after 15 nmol of non-labeled compound in an HT1080 tumor xenograft. 17
Supplementary Table 9. Biodistribution values of [^{177}Lu]Lu-BCY18469 (in %ID/g) at different time points after injection of 150 pmol in an HT1080 tumor xenograft. 18
Supplementary Table 10. Tumor-to-organ ratios in selected organs of [^{177}Lu]Lu-BCY18469 at different time points after injection of 150 pmol in an HT1080 tumor xenograft. 18
Supplementary Table 11. Uptake values of [^{111}In]In-BCY18469 (in %ID/g) at different time points after injection of 230 pmol in an PC-3 tumor xenograft 19
Supplementary Table 12. PK parameters for BCY18469 in mouse following intravenous bolus administration at 1 mg/kg 19

S1. Supplementary Methods

Bicyclic peptide synthesis: Bicyclic peptide was synthesized on Rink amide MBHA resin using standard Fmoc (9-fluorenylmethyloxycarbonyl) solid-phase peptide synthesis, either by manual coupling (for large scale) or using a Biotage Syro II automated peptide synthesizer (for small scale). Following TFA-based cleavage from the resin, the peptides was precipitated in cold isopropyl ether and centrifuged (3 min at 3000 rpm). The solid was washed with isopropyl ether for two additional times, dried under vacuum for 2 hours and dissolved in 40:60 acetonitrile/water. The crude peptide (at ~1 mM concentration) was then cyclized with 1.0 equiv of TATA scaffold, using ammonium bicarbonate in water (1.0 M) as a base to adjust to pH 8. Completion of cyclization was determined by LC−MS. Once complete, the cyclization reaction was quenched using a cysteine solution (10 equiv. with respect to the peptide), and the solution was adjusted to pH 7 using 1M HCl before lyophilization. Afterwards, the residue was dissolved in an appropriate solvent and purified by RP-HPLC. Peptide fractions of sufficient purity and the correct molecular weight (verified by either MALDI-TOF and HPLC or LC−MS) were pooled and lyophilized.

HPLC and radioHPLC analyses: HPLC analyses were carried out (Method A) in an Agilent 1100 series (Agilent Technologies) equipped with a Chromolith RP-18e (100 × 4.6 mm, 2 µm, 130Å – Merck) column. Alternatively (Method B) HPLC analyses were also carried out on an Agilent 1290 UPLC (Agilent Technologies) equipped with an ACQUITY UPLC CSH C18 (150 × 2.1 mm, 1.7µm, 130Å – Agilent). UV absorbance was measured at 220 and/or 254 nm. RadioHPLC measurements for the ^{68}Ga and ^{177}Lu-labeled compound were carried out in the same HPLC system (Agilent 1100), equipped with a RAMONA Star radiodetector (Elysia Raytest). For the ^{111}In-labeled compound, radioHPLC was performed in an Agilent 1100 series (Agilent Technologies) equipped with a Jupiter C18 (150 × 2.6 mm, 5 µm, 300Å– Phenomenex) column and a Raytest Gabi radiodetector (Elysia Raytest). In all cases, analyses were performed using linear gradients of 0.1% TFA in MiliQ-H_2O (A) and 0.1% TFA, 10% MiliQ-H_2O in MeCN (B), with flow rates of 0.4 – 1 mL/min.

Radio TLC analyses: Radio TLC for ^{68}Ga and ^{177}Lu-labeled compounds was performed on iTLC glass microfiber paper (iTLC-SG, Agilent) employing 0.5M $NH_4OAc_{(aq.)}$:DMF (1:1, v/v) as the eluent. For the ^{111}In compound, iTLC was developed using 0.1 mM $NH_4OAc_{(aq.)}$ containing 25 nM of EDTA. TLC plates were read for 5 minutes on a miniGITA Star instrument (Elysia Raytest) or a Cyclone Plus (Perkin Elmer) phosphor imager.

MALDI-TOF/MS analysis: MALDI-TOF/MS was performed on a Daltonics Microflex system (Bruker Daltonics). On a MALDI target, a droplet (1 µL) of a ~0.5 mg/mL solution of the compound was mixed with a droplet (1 µL) of a saturated α-cyano-4-hydroxycinnamic acid (ACCA) solution. The compound was detected as $[M]^+$, $[M \pm H]^+$ and/or $[M + Na]^+$ ions.

Mouse PK study LC-MS: LC-MS/MS analysis was carried out using selected reaction monitoring on a Sciex Triple Quad 6500 Plus with an electrospray ionization source. Chromatographic separation was performed using an ACQUITY UPLC Protein BEH C4 (300Å 1.7 µm 2.1 × 50 mm) column at a temperature of 50°C and flow rate of 0.6 ml/min. Mobile phases of 0.1% formic acid in 95:5 v/v water/acetonitrile and 0.1% formic acid in 95:5 v/v acetonitrile/water were used. Glyburide ($[M+H]^+$ m/z: 494.20) was used as the internal standard for quantification of BCY18469 ($[M+5H]^{5+}$ m/z: 717.10).

S2. Supplementary Figures

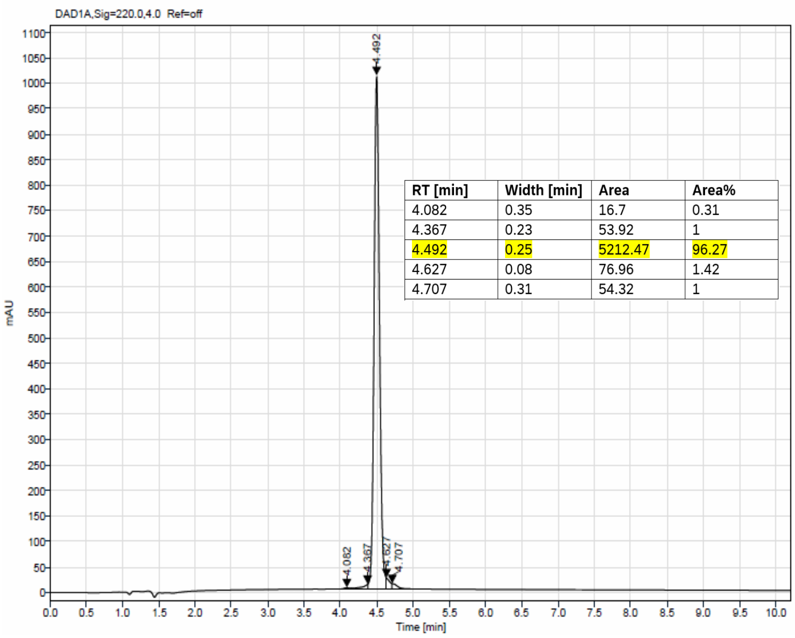

RT [min]	Width [min]	Area	Area%
4.082	0.35	16.7	0.31
4.367	0.23	53.92	1
4.492	0.25	5212.47	96.27
4.627	0.08	76.96	1.42
4.707	0.31	54.32	1

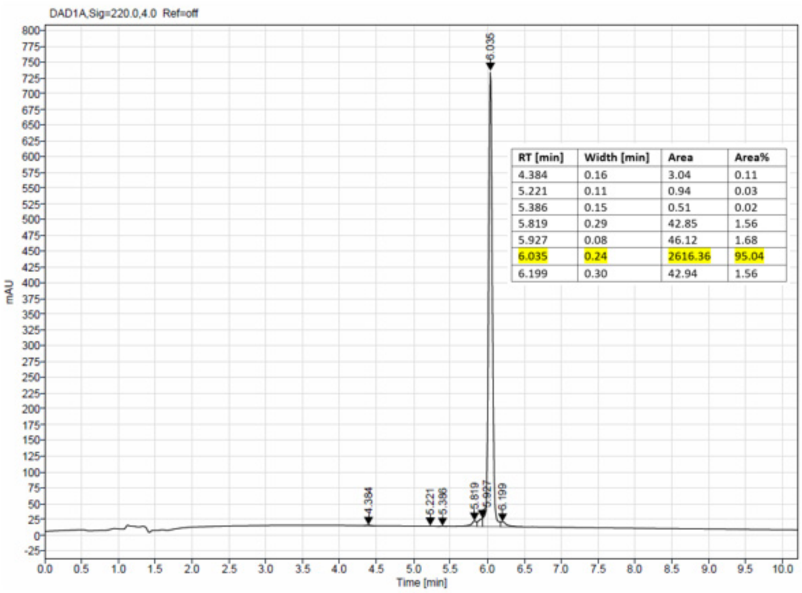

RT [min]	Width [min]	Area	Area%
4.384	0.16	3.04	0.11
5.221	0.11	0.94	0.03
5.386	0.15	0.51	0.02
5.819	0.29	42.85	1.56
5.927	0.08	46.12	1.68
6.035	0.24	2616.36	95.04
6.199	0.30	42.94	1.56

Supplementary Figure 1. HPLC analysis of chemically synthesized and purified BCY18469 (top) and BCY26443 (bottom). Highlighted values correpond to desired product.

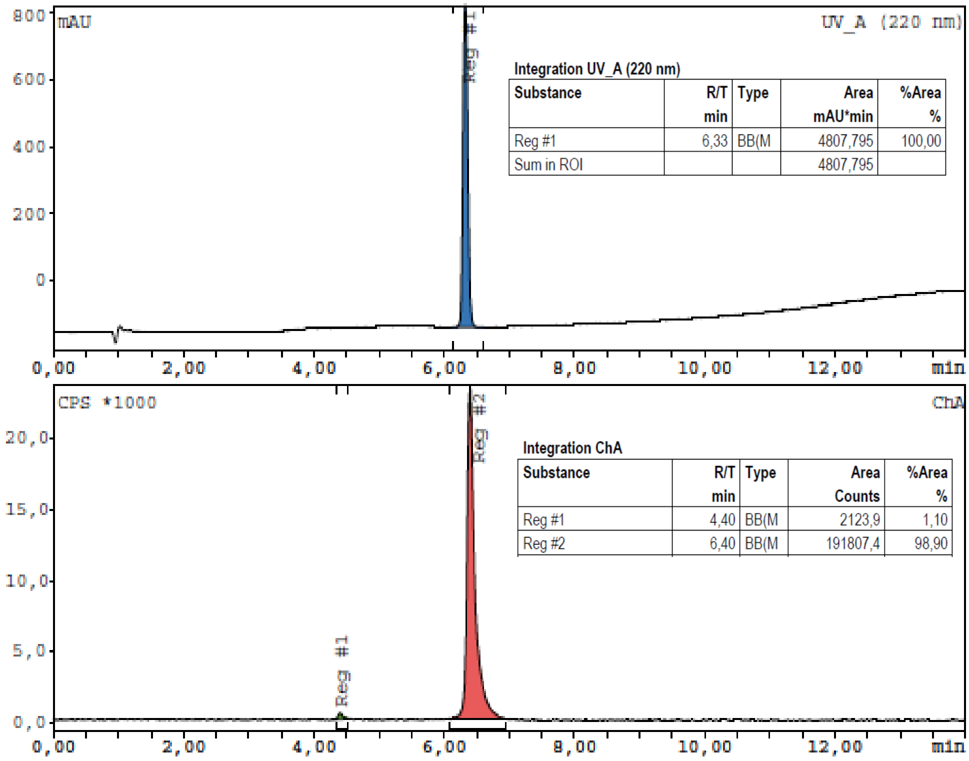

Supplementary Figure 2. HPLC UV-chromatogram (top – UV at 220 nm) and radio HPLC (bottom) of a 'spiked' sample of [^{68}Ga]Ga-BCY18469. Radiolabeled compound was co-injected with 10 µL of 1 mg/mL solution of non-labeled BCY18469.

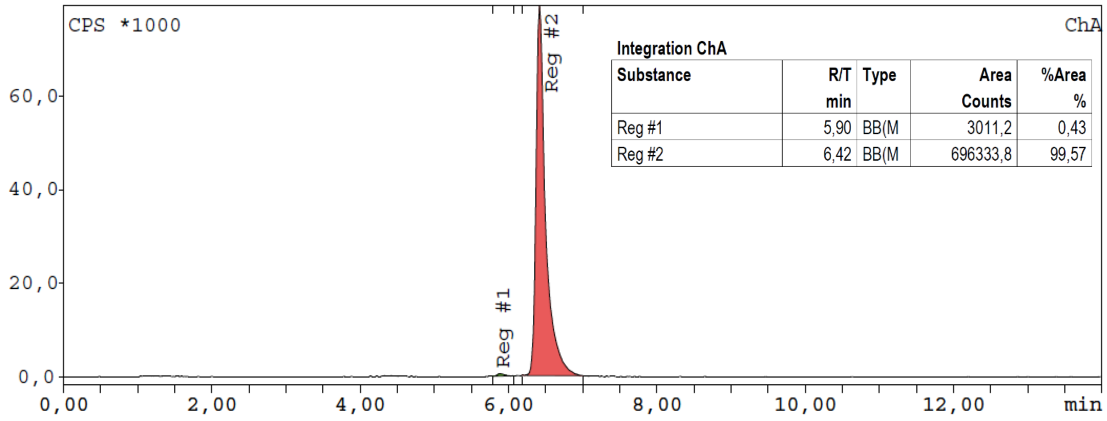

Supplementary Figure 3. Radio HPLC of [^{177}Lu]Lu-BCY18469.

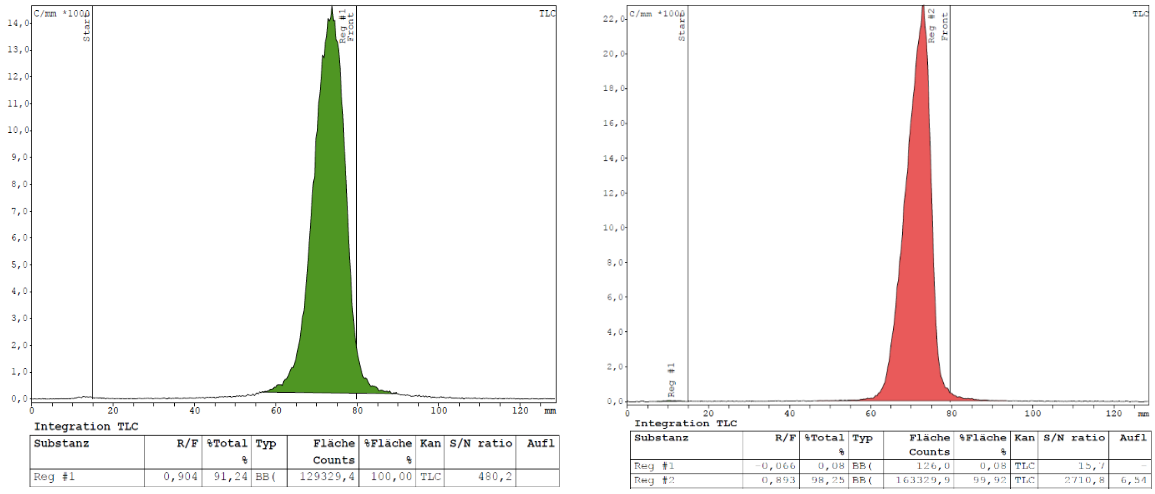

Supplementary Figure 4. Radio iTLC analysis of [^{68}Ga]Ga-BCY18469 (left) and [^{177}Lu]Lu-BCY18469 (right).

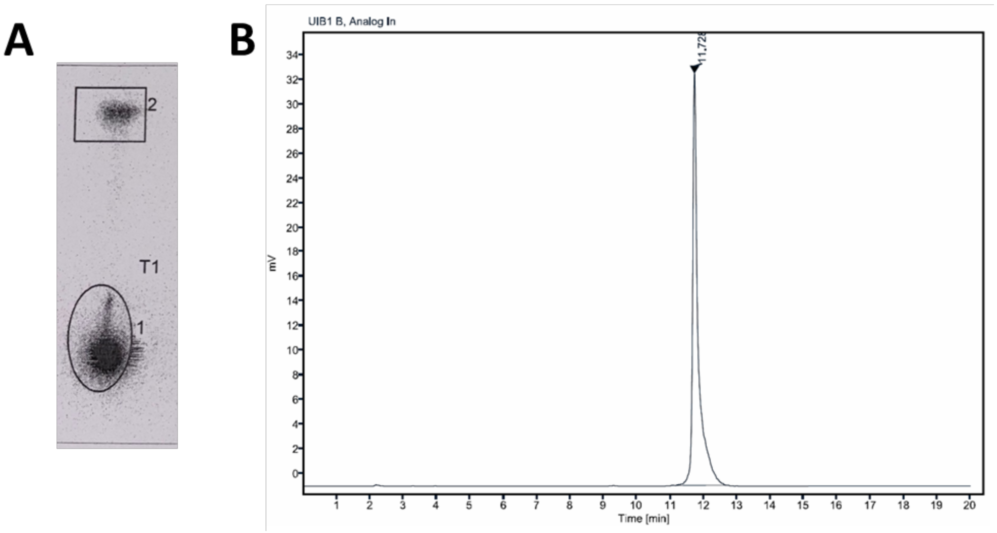

Supplementary Figure 5. A. Phosphor image of radio iTLC of [^{111}In]In-BCY18469 reaction mixture before purification. Percentage at origin 91.8%. **B.** HPLC chromatogram of [^{111}In]In-BCY18469 post purification, purity = 100%.

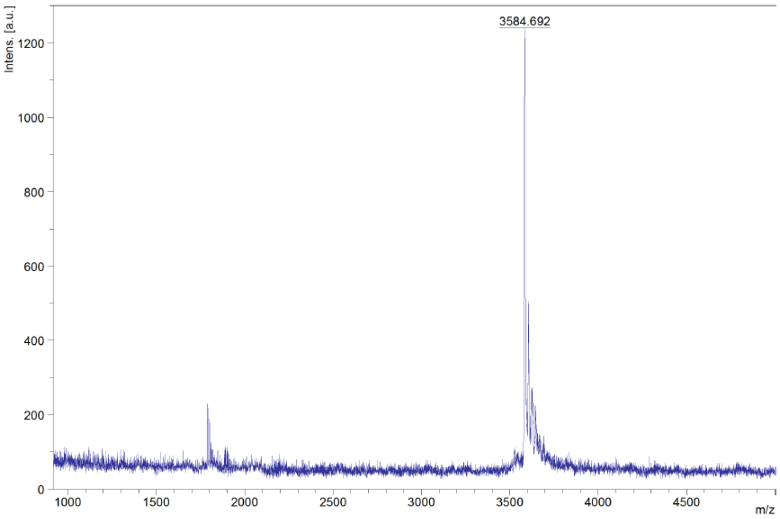

Supplementary Figure 6. MALDI-TOF spectrum of BCY18469 using 2,5-Dihydroxybenzoic acid as a matrix. MS cal.: 3581.11, MS observed: $[M + 3H]^+$ = 3854.69

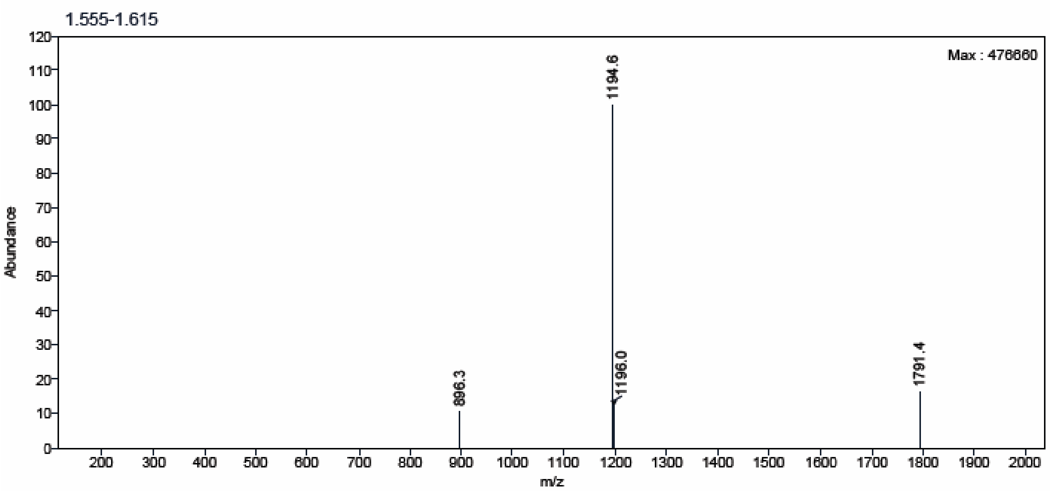

Signal Name MS1 +TIC SCAN ESI Frag=135V Gain=1.0

Peak Retention Time 1.554

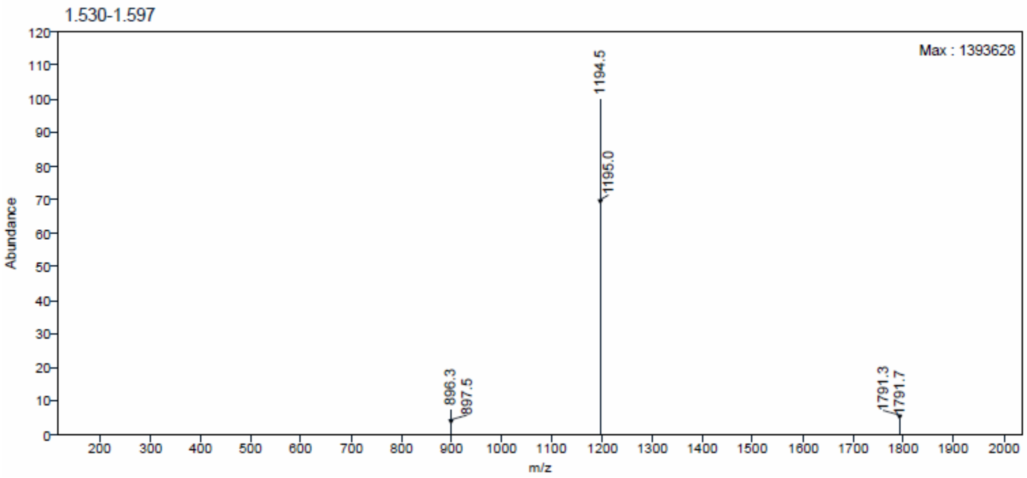

Supplementary Figure 7. Top: ESI-MS spectrum of BCY18469 MS cal.: 3581.11, MS observed: [M + 2H]2^{+} = 1791.55, [M + 3H]3^{+} = 1194.70. Bottom: ESI-MS spectrum of BCY26443. MS cal.: 3581.11; MS observed: [M + 2H]2+ = 1791.3, [M + 3H]3+ = 1194.5

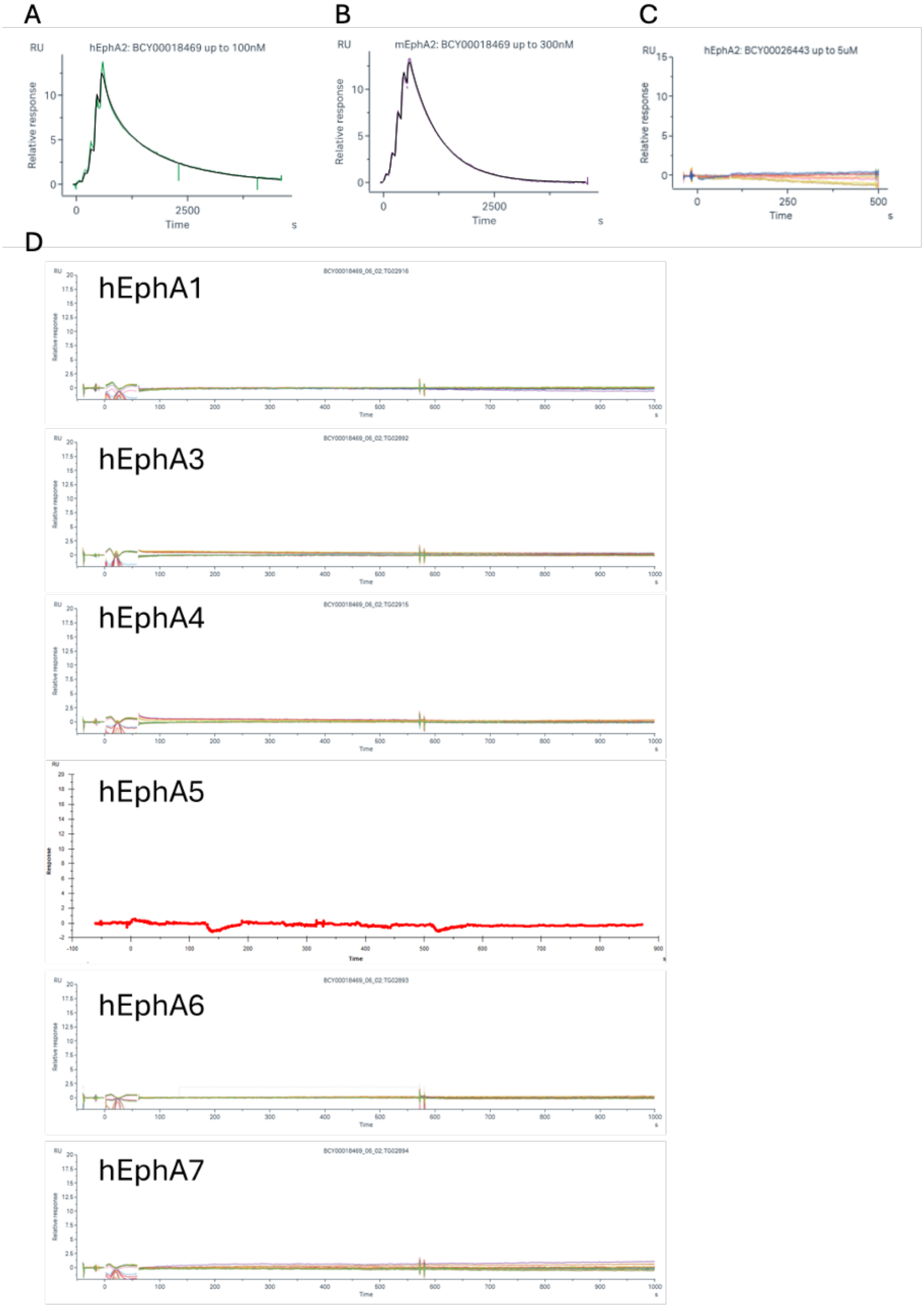

Supplementary Figure 8. (A) Exemplary SPR sensograms of BCY18469 binding to human EphA2 and (B) mouse EphA2. (C) non-binder BCY26443 shows no binding to human EphA2 protein up to 5 μM peptide. (D) BCY18469 shows no binding up to 5 μM when assessed against panel of closely related homologues of EphA2 (EphA1, EphA3, EphA4, EphA5, EphA6, EphA7).

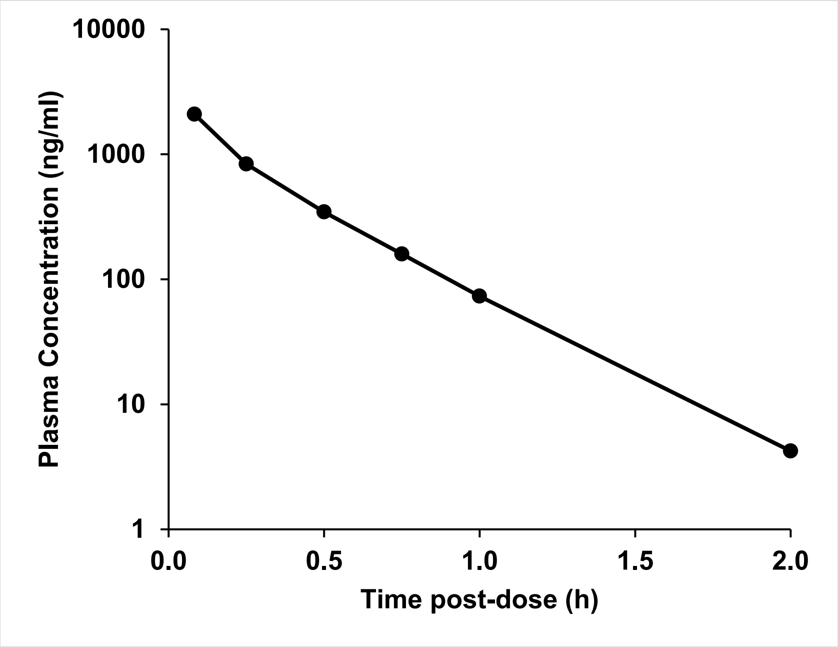

Supplementary Figure 9. Plasma concentration –time profile following intravenous administration of BCY18469 (1 mg/kg).

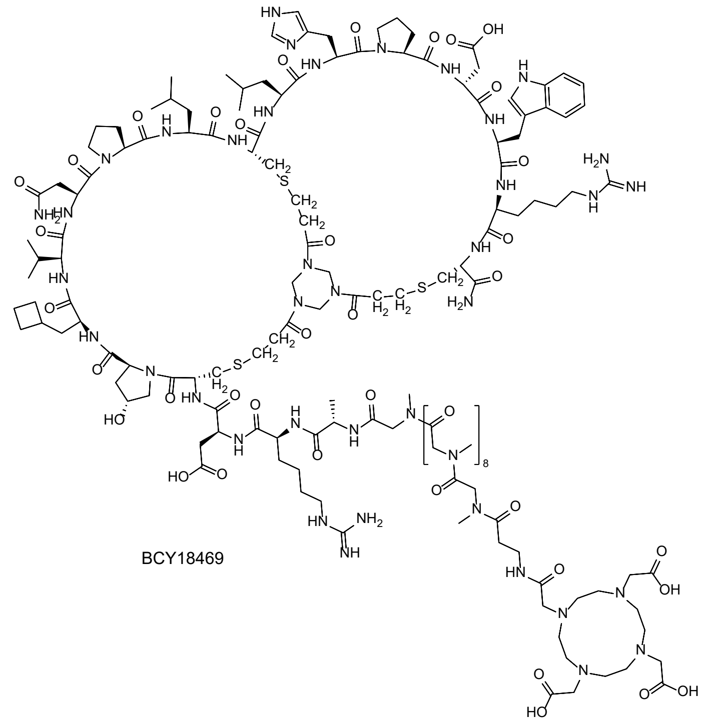

BCY18469

[-DOTA]-[B-Ala]-[Sar10]-Ala-[HArg]-Asp-Cys-[HyP]-[Cba]-Val-Asn-Pro-Leu-Cys-Leu-His-Pro-[dAsp]-Trp-[HArg]-Cys-CONH2

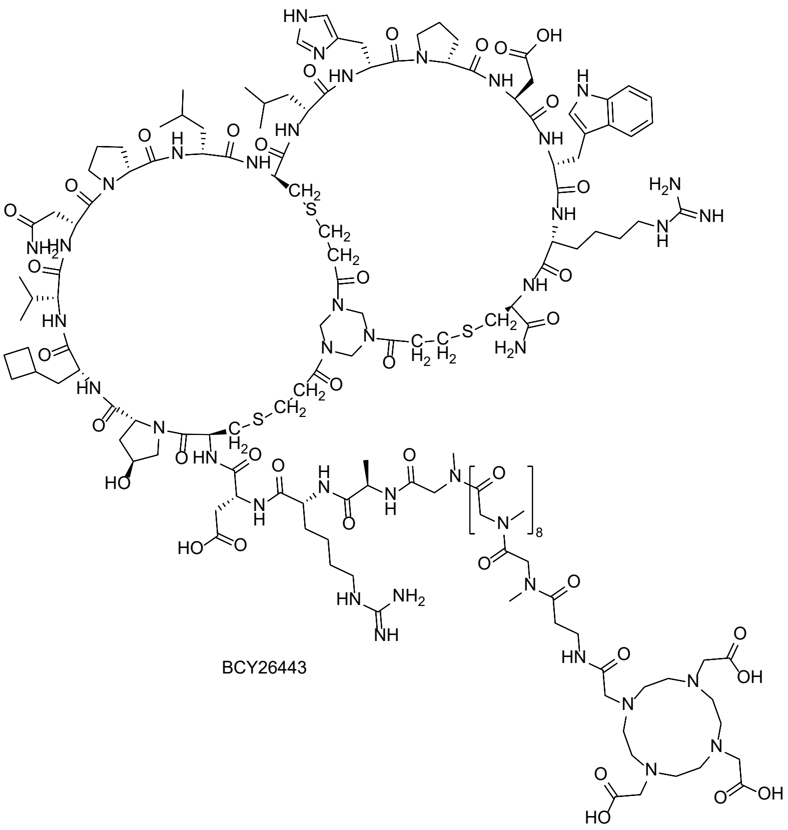

BCY26443

[DOTA]-[B-Ala]-[Sar10]-[dAla]-[dHArg]-[dAsp]-[dCys]-[dHyP]-[dCba]-[dVal]-[dAsn]-[dPro]-[dLeu]-[dCys]-[dLeu]-[dHis]-[dPro]-Asp-[dTrp]-[dHArg]-[dCys]-CONH2

Supplementary Figure 10. Top: Chemical structure and sequence of BCY18469. Bottom: Chemical structure and sequence of BCY26443

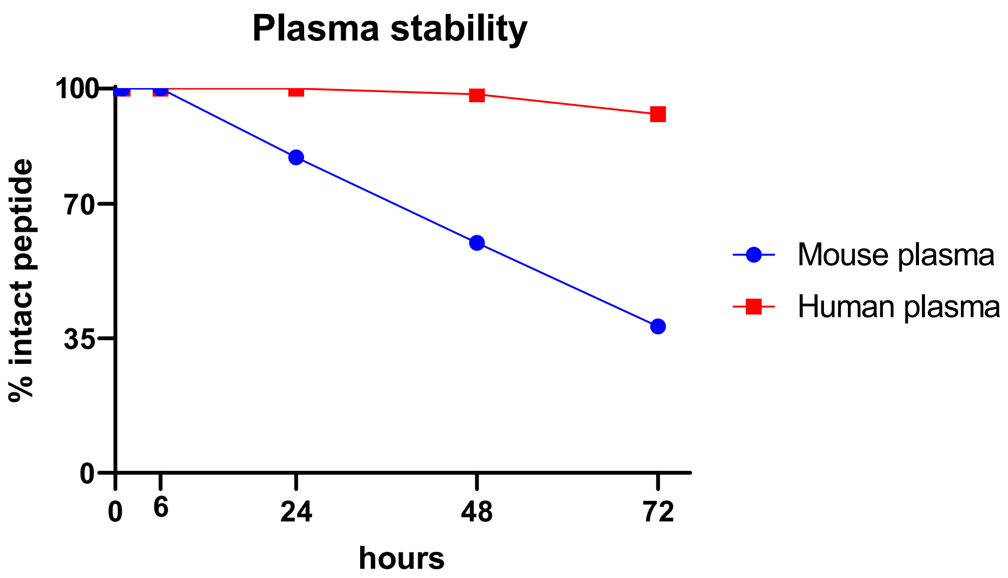

Supplementary Figure 11. *In vitro* stability of [^{177}Lu]Lu-BCY18469 in mouse plasma (blue line) and human plasma (red line). Determined over 72-h incubation by HPLC, following the method previously described (Method A).

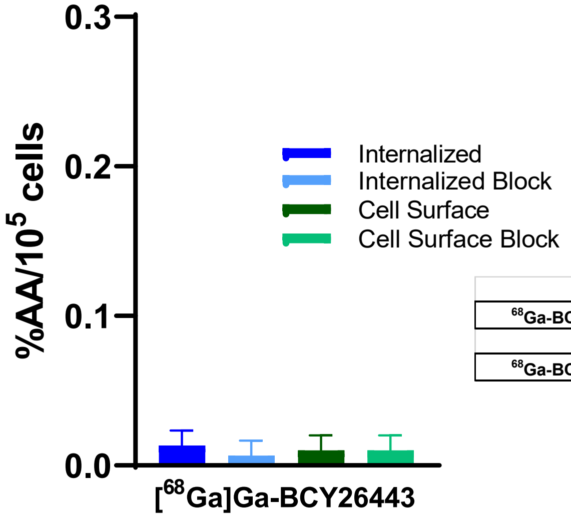

	Cell Surface	Cell Surface B
^{68}Ga-BC26443	0.01 ± 0.01	0.01 ± 0.01
	Internalization	Internalization B
^{68}Ga-BC26443	0.01 ± 0.01	0.01 ± 0.01

Supplementary Figure 12. Cell surface binding and internalization of the non-binding (negative control) bicycle [^{68}Ga]Ga-BCY26443. Specificity of internalization was determined by blocking with an excess of non-labeled compound (10 µM). Data expressed as % applied activity/10^5 cells (n = 3)

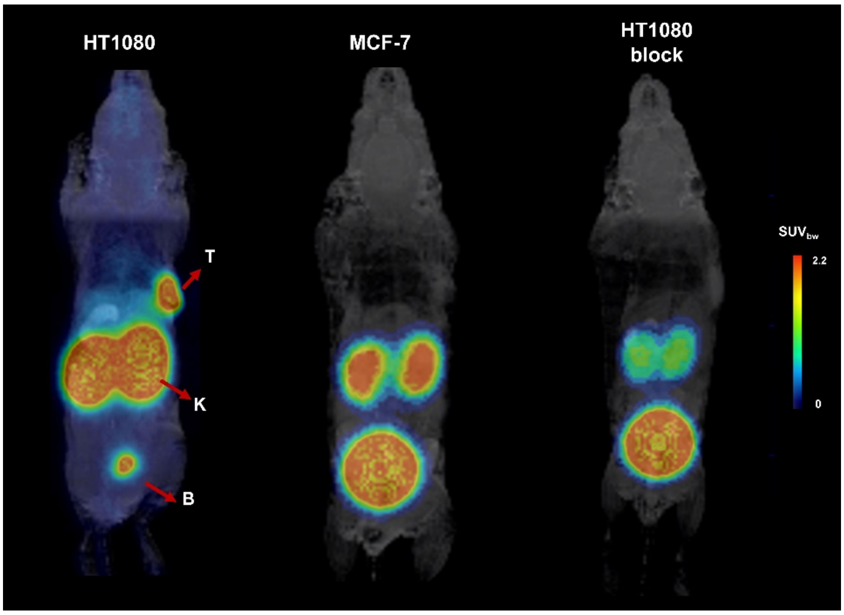

Supplementary Figure 13. PET/MR Maximum Intensity Projections (MIPs) (from left to right) 1 h p.i. of 150 pmol of [^{68}Ga]Ga-BCY18469 in an HT1080 and MCF-7 xenograft, and 1 h p.i. of 300 pmol of [^{68}Ga]Ga-BCY18469 after 15 nmol of BCY18469 in an HT1080 xenograft. T: tumor, K: kidney, B: bladder.

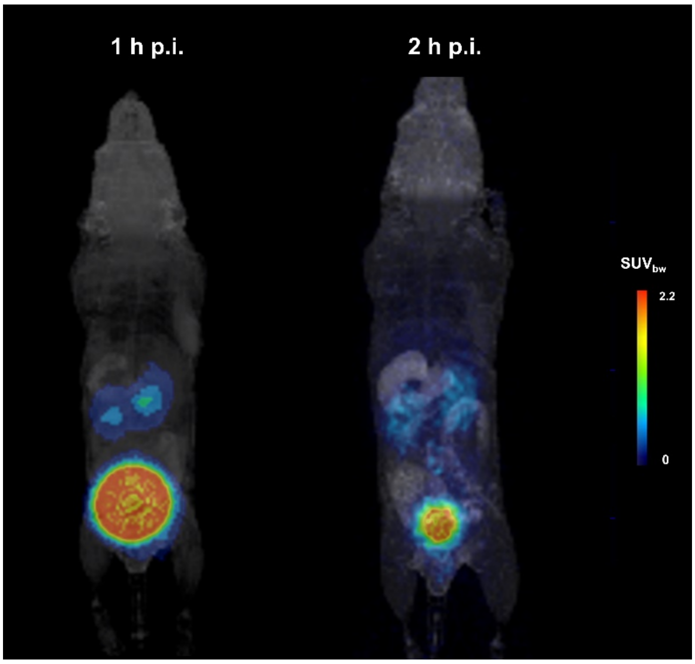

Supplementary Figure 14. PET/MR Maximum Intensity Projection (MIP) (from left to right) 1 h and 2 h p.i. of 150 pmol of non-EphA2 binding [^{68}Ga]Ga-BCY26443 in an HT1080 mouse xenograft.

S3. Supplementary Tables

Supplementary Table 1. Affinity values of BCY18469 obtained from SPR against mouse and human EphA2 and a panel of close homologues of EphA2.

Protein	KD (M)	ka (1/Ms)	kd (1/s)
**Human EphA2, n=2**	1.93E-09 ± 3.54E-10	1.82E+06 ± 4.24E+04	3.52E-03 ± 7.21E-04
**Mouse EphA2, n=2**	3.82E-09 ± 5.59E-10	4.19E+05 ± 9.33E+04	1.57E-03 ± 1.27E-04
**Human EphA1, n=1**	No binding up to 5 µM	-	-
**Human EphA3, n=1**	No binding up to 5 µM	-	-
**Human EphA4, n=1**	No binding up to 5 µM	-	-
**Human EphA5, n=1**	No binding up to 5 µM	-	-
**Human EphA6, n=1**	No binding up to 5 µM	-	-
**Human EphA7, n=1**	No binding up to 5 µM	-	-

Supplementary Table 2. Detailed internalization values for ^{68}Ga and ^{177}Lu-labeled BCY18469. *n* = 3. B: block

	Cell Surface	Cell Surface B	Cell Surface 4°C	Cell Surface B 4°C
**^{177}Lu-BCY18469**	0.62 ± 0.03	0.02 ± 0.01	0.51 ± 0.01	0.05 ± 0.01
**^{68}Ga-BCY18469**	0.53 ± 0.03	0.02 ± 0.01	0.47 ± 0.01	0.03 ± 0.01
	**Internalization**	**Internalization B**	**Internalization 4°C**	**Internalization B 4°C**
**^{177}Lu-BCY18469**	0.13 ± 0.01	0.01 ± 0.01	0.06 ± 0.01	0.01 ± 0.01
**^{68}Ga-BCY18469**	0.18 ± 0.05	0.06 ± 0.00	0.05 ± 0.01	0.01 ± 0.00

Supplementary Table 3. Uptake values of [^{68}Ga]Ga-BCY18469 over 60 min p.i. (in SUV_{bw}) in PET/MRI after injection of 150 pmol in an HT1080 tumor xenograft.

Minutes p.i.	Tumor	Bladder	Kidneys	Heart	Liver	Muscle
0.0	0.0	0.0	0.0	0.0	0.0	0.0
0.1	0.67	0.21	1.64	6.48	1.56	0.08
0.3	0.54	0.25	1.87	4.94	1.45	0.13
0.4	0.53	0.28	1.94	4.11	1.46	0.14
0.5	0.53	0.28	1.93	3.62	1.49	0.16
0.7	0.56	0.27	1.89	3.46	1.53	0.15
0.8	0.56	0.30	1.83	3.18	1.61	0.17
0.9	0.58	0.30	1.75	2.93	1.76	0.19
1.1	0.58	0.30	1.68	2.74	1.76	0.20
1.2	0.61	0.31	1.63	2.59	1.79	0.19
1.3	0.60	0.34	1.59	2.42	1.82	0.22
1.5	0.66	0.37	1.57	2.37	1.73	0.22
1.6	0.67	0.38	1.62	2.26	1.63	0.23
1.7	0.69	0.41	1.70	2.13	1.59	0.24
1.9	0.69	0.40	1.81	2.02	1.49	0.26
2.0	0.70	0.42	1.89	1.94	1.46	0.25
3.0	0.73	0.40	2.29	1.67	1.30	0.27
4.0	0.82	0.40	2.81	1.35	1.13	0.27
5.0	0.91	0.41	3.15	1.16	1.04	0.27

7.0	1.03	0.44	3.54	0.98	0.94	0.28
9.0	1.17	0.51	3.92	0.82	0.86	0.27
11.0	1.27	0.60	4.20	0.74	0.81	0.27
13.0	1.36	0.69	4.42	0.69	0.77	0.25
15.0	1.42	0.76	4.61	0.64	0.74	0.25
20.0	1.51	0.80	4.92	0.58	0.70	0.24
25.0	1.58	0.80	5.29	0.53	0.66	0.22
30.0	1.63	0.79	5.61	0.49	0.64	0.20
35.0	1.67	0.77	5.92	0.45	0.61	0.19
40.0	1.70	0.76	6.18	0.43	0.60	0.18
45.0	1.71	0.75	6.44	0.40	0.59	0.17
60.0	1.71	0.74	6.83	0.35	0.56	0.15

Supplementary Table 4. Uptake values of [^{68}Ga]Ga-BCY18469 over 60 min p.i. (in %ID/g) in PET/MRI after injection of 150 pmol in an HT1080 tumor xenograft.

Minutes p.i.	Tumor	Bladder	Kidneys	Heart	Liver	Muscle
0.0	0.0	0.0	0.0	0.0	0.0	0.0
0.1	3.2	1.0	7.0	31.3	7.6	0.4
0.3	2.6	1.2	8.3	23.9	7.0	0.6
0.4	2.6	1.3	8.7	19.9	7.0	0.7
0.5	2.6	1.4	8.7	17.5	7.2	0.8
0.7	2.7	1.3	8.5	16.7	7.4	0.7
0.8	2.7	1.4	8.2	15.4	7.8	0.8
0.9	2.8	1.4	7.8	14.2	8.5	0.9
1.1	2.8	1.4	7.6	13.2	8.5	1.0
1.2	3.0	1.5	7.3	12.5	8.7	0.9
1.3	2.9	1.6	7.1	11.7	8.8	1.0
1.5	3.2	1.8	7.0	11.4	8.3	1.1
1.6	3.2	1.9	7.2	10.9	7.9	1.1
1.7	3.3	2.0	7.6	10.3	7.7	1.1
1.9	3.3	2.0	8.2	9.7	7.2	1.3
2.0	3.4	2.0	8.7	9.4	7.1	1.2
3.0	3.5	1.9	10.7	8.0	6.3	1.3
4.0	3.9	1.9	13.3	6.5	5.5	1.3
5.0	4.4	2.0	15.1	5.6	5.0	1.3
7.0	5.0	2.1	16.9	4.7	4.6	1.3
9.0	5.7	2.5	18.7	4.0	4.2	1.3
11.0	6.1	2.9	20.1	3.6	3.9	1.3
13.0	6.5	3.4	21.3	3.3	3.7	1.2
15.0	6.9	3.7	22.3	3.1	3.6	1.2
20.0	7.3	3.9	23.9	2.8	3.4	1.1
25.0	7.6	3.9	25.7	2.6	3.2	1.1
30.0	7.9	3.8	27.3	2.4	3.1	1.0
35.0	8.1	3.7	28.7	2.2	3.0	0.9
40.0	8.2	3.7	29.9	2.1	2.9	0.9

45.0	8.2	3.6	31.1	1.9	2.8	0.8
60.0	8.3	3.6	32.9	1.7	2.7	0.7

Supplementary Table 5. Uptake values of [^{68}Ga]Ga-BCY18469 over 60 min p.i. (in SUV_{bw}) in PET/MRI after injection of 150 pmol in a MCF-7 (EphA2-negative) tumor xenograft.

Minutes p.i.	Tumor	Bladder	Kidneys	Heart	Liver	Muscle
0.0	0.0	0.0	0.0	0.0	0.0	0.0
0.1	0.34	0.48	1.06	4.10	1.75	0.17
0.3	0.53	0.43	1.69	2.92	1.59	0.31
0.4	0.73	0.67	2.07	6.06	3.01	0.31
0.5	0.69	0.57	2.83	3.93	2.34	0.38
0.7	0.67	0.59	2.76	3.46	2.43	0.38
0.8	0.72	0.56	2.84	2.89	2.42	0.38
0.9	0.67	0.41	2.97	2.78	2.41	0.39
1.1	0.71	0.48	3.12	2.74	2.22	0.41
1.2	0.77	0.50	3.24	2.66	2.16	0.46
1.3	0.66	0.44	3.49	2.53	1.95	0.45
1.5	0.65	0.45	3.70	2.18	1.86	0.42
1.6	0.64	0.48	3.91	2.17	1.81	0.46
1.7	0.65	0.44	4.03	2.11	1.70	0.42
1.9	0.71	0.43	4.12	2.07	1.67	0.42
2.0	0.66	0.49	4.37	2.02	1.64	0.42
3.0	0.67	0.52	4.95	1.73	1.40	0.45
4.0	0.69	0.54	6.10	1.44	1.12	0.45
5.0	0.68	1.74	6.84	1.18	0.98	0.44
7.0	0.65	9.97	6.73	0.97	0.80	0.42
9.0	0.63	24.47	5.69	0.81	0.66	0.39
11.0	0.59	34.23	4.74	0.68	0.56	0.36
13.0	0.55	33.70	3.95	0.57	0.51	0.32
15.0	0.53	26.30	3.40	0.51	0.46	0.30
20.0	0.48	19.23	2.90	0.43	0.38	0.26
25.0	0.39	26.02	2.68	0.32	0.30	0.20
30.0	0.34	28.20	2.58	0.26	0.25	0.16
35.0	0.28	30.15	2.47	0.20	0.21	0.13
40.0	0.24	31.40	2.42	0.17	0.18	0.10
45.0	0.21	31.23	2.37	0.15	0.16	0.08
60.0	0.16	31.47	2.34	0.11	0.12	0.06

Supplementary Table 6. Uptake values of [^{68}Ga]Ga-BCY18469 over 60 min p.i. (in %ID/g) in PET/MRI after injection of 150 pmol in a MCF-7 (EphA2-negative) tumor xenograft.

Minutes p.i.	Tumor	Bladder	Kidneys	Heart	Liver	Muscle
0.0	0.0	0.0	0.0	0.0	0.0	0.0
0.1	2.1	2.8	6.1	24.1	10.3	0.5
0.3	1.4	2.5	9.6	17.2	9.4	0.8
0.4	2.9	3.9	11.6	35.7	17.7	1.2
0.5	2.3	3.4	15.9	23.1	13.8	1.3
0.7	2.2	3.4	15.9	20.3	14.3	1.6
0.8	2.7	3.3	15.9	17.0	14.2	1.7
0.9	2.4	2.4	16.5	16.4	14.2	2.0
1.1	2.7	2.8	17.3	16.1	13.0	2.2
1.2	2.6	3.0	18.4	15.6	12.7	2.0
1.3	2.5	2.6	19.4	14.9	11.5	2.2
1.5	2.4	2.6	20.4	12.8	11.0	2.2
1.6	2.4	2.8	21.5	12.8	10.6	2.3
1.7	2.3	2.6	22.1	12.4	10.0	2.2
1.9	2.4	2.5	22.8	12.2	9.8	2.5
2.0	2.5	2.9	24.2	11.9	9.6	2.4
3.0	2.5	3.1	27.1	10.2	8.3	2.3
4.0	2.5	3.2	33.1	8.5	6.6	2.4
5.0	2.5	10.2	37.7	6.9	5.8	2.4
7.0	2.4	58.6	38.4	5.7	4.7	2.2
9.0	2.4	143.9	33.2	4.8	3.9	2.0
11.0	2.2	201.4	27.5	4.0	3.3	1.8
13.0	2.1	198.2	22.7	3.4	3.0	1.7
15.0	2.1	154.7	19.3	3.0	2.7	1.5
20.0	1.8	113.1	16.5	2.5	2.2	1.3
25.0	1.6	153.1	15.3	1.9	1.8	1.0
30.0	1.4	165.9	14.8	1.5	1.5	0.8
35.0	1.3	177.3	14.2	1.2	1.2	0.7
40.0	1.1	184.7	13.9	1.0	1.1	0.5
45.0	1.0	183.7	13.7	0.9	0.9	0.4
60.0	0.9	185.1	13.6	0.6	0.7	0.3

Supplementary Table 7. Uptake values of [^{68}Ga]Ga-BCY18469 over 60 min p.i. (in SUV_{bw}) in PET/MRI after injection of 300 pmol after 15 nmol of non-labeled compound (block) in an HT1080 tumor xenograft.

Minutes p.i.	Tumor	Bladder	Kidneys	Heart	Liver	Muscle
0.0	0.0	0.0	0.0	0.0	0.0	0.0
0.1	0.32	0.29	1.19	2.83	1.21	0.03
0.3	0.35	0.32	1.71	2.36	1.35	0.12
0.4	0.67	0.42	2.35	4.48	2.32	0.21
0.5	0.52	0.40	2.47	2.95	2.13	0.28
0.7	0.55	0.45	2.55	2.67	2.16	0.33
0.8	0.60	0.48	2.75	2.51	2.05	0.30
0.9	0.56	0.49	2.72	2.43	1.93	0.35
1.1	0.61	0.54	3.00	2.22	1.82	0.35
1.2	0.57	0.63	3.18	2.14	1.74	0.36
1.3	0.63	0.54	3.16	2.04	1.69	0.36
1.5	0.61	0.58	3.35	1.97	1.63	0.38
1.6	0.60	0.56	3.41	1.88	1.55	0.42
1.7	0.59	0.54	3.52	1.84	1.52	0.40
1.9	0.63	0.54	3.49	1.80	1.47	0.44
2.0	0.58	0.53	3.68	1.79	1.39	0.35
3.0	0.67	0.84	4.87	1.62	1.27	0.40
4.0	0.66	5.92	5.22	1.47	1.08	0.41
5.0	0.62	12.08	4.37	1.24	0.95	0.40
7.0	0.58	17.73	3.45	1.05	0.81	0.37
9.0	0.55	21.83	2.94	0.90	0.70	0.35
11.0	0.53	24.11	2.68	0.81	0.62	0.31
13.0	0.49	28.00	2.35	0.72	0.58	0.29
15.0	0.46	29.75	2.07	0.66	0.53	0.27
20.0	0.42	31.67	1.85	0.56	0.45	0.23
25.0	0.38	33.49	1.78	0.46	0.37	0.19
30.0	0.34	34.26	1.59	0.39	0.31	0.15
35.0	0.31	34.41	1.50	0.32	0.28	0.13
40.0	0.27	33.18	1.39	0.28	0.25	0.10
45.0	0.25	32.22	1.33	0.25	0.22	0.09
60.0	0.21	30.60	1.24	0.21	0.19	0.07

Supplementary Table 8. Uptake values of [^{68}Ga]Ga-BCY18469 over 60 min p.i. (in %ID/g) in PET/MRI after injection of 300 pmol after 15 nmol of non-labeled compound (block) in an HT1080 tumor xenograft.

Minutes p.i.	Tumor	Bladder	Kidneys	Heart	Liver	Muscle
0.0	0.0	0.0	0.0	0.0	0.0	0.0
0.1	2.1	1.9	7.1	18.7	8.0	0.2
0.3	2.3	2.1	10.9	15.6	9.0	0.8
0.4	4.4	2.8	15.2	29.7	15.4	1.4
0.5	3.5	2.6	16.1	19.5	14.1	1.8
0.7	3.6	3.0	16.5	17.7	14.3	2.2
0.8	3.9	3.2	17.2	16.6	13.6	2.0
0.9	3.7	3.3	17.5	16.1	12.8	2.3
1.1	4.1	3.6	19.3	14.7	12.1	2.3
1.2	3.7	4.2	20.3	14.2	11.5	2.4
1.3	4.2	3.6	20.5	13.5	11.2	2.4
1.5	4.1	3.8	21.7	13.0	10.8	2.5
1.6	3.9	3.7	21.8	12.5	10.2	2.8
1.7	3.9	3.6	22.8	12.2	10.0	2.6
1.9	4.2	3.6	22.7	11.9	9.7	2.9
2.0	3.9	3.5	23.9	11.9	9.2	2.3
3.0	4.4	5.6	31.2	10.8	8.4	2.7
4.0	4.4	39.2	34.2	9.8	7.2	2.7
5.0	4.1	80.0	28.8	8.2	6.3	2.7
7.0	3.9	117.4	22.5	6.9	5.4	2.4
9.0	3.7	144.5	19.2	6.0	4.6	2.3
11.0	3.5	159.6	17.3	5.3	4.1	2.1
13.0	3.2	185.4	14.7	4.8	3.8	1.9
15.0	3.1	197.0	13.1	4.4	3.5	1.8
20.0	2.8	209.7	12.0	3.7	3.0	1.5
25.0	2.5	221.8	11.4	3.1	2.5	1.2
30.0	2.2	226.9	10.2	2.6	2.1	1.0
35.0	2.0	227.8	9.6	2.1	1.8	0.8
40.0	1.8	219.7	8.9	1.9	1.6	0.7
45.0	1.6	213.4	8.7	1.7	1.4	0.6
60.0	1.4	202.7	8.1	1.4	1.3	0.5

Supplementary Table 9. Biodistribution values of [^{177}Lu]Lu-BCY18469 (in %ID/g) at different time points after injection of 150 pmol in an HT1080 tumor xenograft.

	**1 h p.i.**			**2 h p.i.**			**6 h p.i.**			**24 h p.i.**		
	**Mean**	**SD**	**n**	**Mean**	**SD**	**n**	**Mean**	**SD**	**n**	**Mean**	**SD**	**n**
**Blood**	0.2	0.06	3	0.03	0.01	3	0.02	0	3	0.01	0	3
**Heart**	0.1	0.03	3	0.03	0	3	0.05	0.01	3	0.03	0.01	3
**Lung**	0.7	0.3	3	0.19	0.04	3	0.28	0.09	3	0.13	0.05	3
**Spleen**	0.6	0.1	3	0.16	0.02	3	0.4	0.01	3	0.31	0.08	3
**Liver**	0.25	0	3	0.15	0.02	3	0.6	0.03	3	0.53	0.15	3
**Kidneys**	65.3	15.1	3	47.23	1.68	3	62.6	15.28	3	54.86	17.12	3
**Muscle**	0.06	0	3	0.02	0	3	0.03	0.01	3	0.02	0	3
**Intestine**	0.34	0.1	3	0.16	0.1	3	0.15	0.01	3	0.1	0.01	3
**Brain**	0	0	3	0.01	0	3	0.01	0	3	0	0	3
**Tumor**	19.5	3.5	3	9.99	1.24	3	5.56	1.84	3	1.77	0.51	3
**Tail**	1.8	0.6	3	0.7	0.42	3	0.83	0.47	3	0.38	0.21	3
**Bone**	0.3	0.1	3	0.21	0.15	3	0.56	0.02	3	0.44	0.08	3

Supplementary Table 10. Tumor-to-organ ratios in selected organs of [^{177}Lu]Lu-BCY18469 at different time points after injection of 150 pmol in an HT1080 tumor xenograft.

	**1 h p.i.**			**2 h p.i.**			**6 h p.i.**			**24 h p.i.**		
	**Mean**	**SD**	**n**	**Mean**	**SD**	**n**	**Mean**	**SD**	**n**	**Mean**	**SD**	**n**
**Blood**	97.5	26.28	3	333	78.33	3	278	92	3	177	51	3
**Heart**	195	52.55	3	333	41.33	3	111.2	41.25	3	59	23.56	3
**Lung**	27.86	10.12	3	52.58	8.86	3	19.86	8.62	3	13.62	5.94	3
**Spleen**	32.5	6.74	3	62.44	8.73	3	13.9	4.61	3	5.71	2.03	3
**Liver**	78	14	3	66.6	9.45	3	9.27	3.09	3	3.34	1.23	3
**Kidneys**	0.3	0.07	3	0.21	0.03	3	0.09	0.03	3	0.03	0.01	3
**Muscle**	325	58.33	3	499.5	62	3	185.33	81.93	3	88.5	25.5	3
**Intestine**	57.35	15.26	3	62.44	32.14	3	37.07	12.43	3	17.7	5.28	3
**Tail**	10.83	3.15	3	14.27	6.91	3	6.7	4.36	3	4.66	2.76	3

Supplementary Table 11. Uptake values of [^{111}In]In-BCY18469 (in %ID/g) at different time points after injection of 230 pmol in an PC-3 tumor xenograft.

	1 h p.i.			24 h p.i.		
	Mean	SD	n	Mean	SD	n
**Bladder**	206.7	119.2	3	4.2	0.5	3
**Kidneys**	32.0	3.4	3	17.0	2.5	3
**Heart**	0.03	0.01	3	0.03	0.02	3
**Liver**	0.13	0.09	3	0.05	0.03	3
**Lungs**	0.12	0.02	3	0.01	0.00	3
**Muscle**	0.27	0.25	3	0.02	0.02	3
**Spleen**	0.27	0.19	3	0.40	0.19	3
**Tail**	0.28	0.09	3	0.07	0.01	3
**Tumor**	5.7	1.5	3	2.3	0.5	3

Supplementary Table 12. PK parameters for BCY18469 in mouse following intravenous bolus administration at 1 mg/kg. Abbreviations: **AUC** = area under the plasma concentration time curve, **CLp** = plasma clearance, $\mathbf{C_{max}}$ = maximum mean plasma concentration, $\mathbf{t_{1/2}}$ = half-life, $\mathbf{Vd_{ss}}$ = volume of distribution at steady state

PK Parameter	BCY18469
C_{max} (ng/mL)	3322
$t_{1/2}$ (h)	0.24
Vd_{ss} (L/kg)	0.39
CLp (mL/min/kg)	23.9
$AUC_{0\text{-}last}$ (ng.h/mL)	700
$AUC_{0\text{-}inf}$ (ng.h/mL)	701
